# How Is Registered Nursing Time Used? A Multicentre Observational Study of Nursing Activities, Scope of Practice, and Opportunities for Workforce Redesign in Medical–Surgical Wards in Italy

**DOI:** 10.3390/nursrep16100372

**Published:** 2026-10-20

**Authors:** Dhurata Ivziku, Luca Vollero, Viviana Alciati, Federica Maria Pia Ferramosca, Viviana Cotichini, Francesca Reato, Marzia Lommi, Daniela Tartaglini

**Affiliations:** 1 Department of Health Professions, Fondazione Policlinico Universitario Campus Bio-Medico, 00128 Rome, Italy; d.tartaglini@policlinicocampus.it; 2 Faculty of Health Sciences, AAB College, 10000 Pristina, Kosovo; 3 Faculty of Biomedical Engeneering, University Campus Bio-Medico, 00128 Rome, Italy; l.vollero@unicampus.it; 4 Department of Biomedicine and Prevention, University of Rome Tor Vergata, 00133 Rome, Italy; marzia.lommi@uniroma2.eu; 5 Department of Anesthesia and Reanimation, ASL Lecce, 73100 Lecce, Italy; federica_ferramosc@hotmail.it; 6 Department of Health Professions, ARES 118, 00149 Rome, Italy; vivianacotichini@live.it; 7 Bachelor’s Degree Program in Nursing, University of Insubria, 21100 Varese, Italy; francesca.reato@uninsubria.it; 8 Research Unit in Nursing Science, Faculty of Medicine and Surgery, University Campus Bio-Medico, 00128 Rome, Italy

**Keywords:** nursing workload, time and motion studies, nursing activities, scope of practice, skill mix, workforce planning, medical–surgical nursing, hospital, nurse assistant

## Abstract

**Background/Objectives**: Nursing workforce shortages and increasing care complexity have intensified the need to understand how registered nurses allocate their working time and whether nursing expertise is used where it adds the greatest clinical value. This study empirically measured the distribution, frequency, and duration of observed nursing activities and examined differences across participating hospitals and medical–surgical units in Italy. **Methods**: A multicentre observational study was conducted across 13 medical and surgical units in five Italian teaching hospitals. Registered nurses were observed during complete morning and afternoon shifts over a three-week period. Activities were classified as direct care, indirect care, organisational activities, or non-patient care. Time allocation, activity frequency, and duration were examined across units and hospitals. The Enacted Scope of Practice model, Role Theory, and Job Demands–Resources Theory informed interpretation of the findings but were not used to classify individual activities. **Results**: Overall, 45% of cumulative recorded activity time was devoted to direct care, 24% to indirect care, 10% to organisational activities, and 21% to non-patient care. Direct care accounted for 51% of recorded activity time in medical units and 41% in surgical units, whereas indirect care represented 17% and 28%, respectively. Considerable hospital-level variability was observed, particularly for indirect, organisational, and non-patient care activities. Direct care was also the most frequent activity category, while organisational activities were less frequent but comparatively time-intensive. **Conclusions**: Nursing work extends substantially beyond bedside care and includes clinically relevant indirect and organisational activities. Interpreted through the Enacted Scope of Practice framework, the findings identify areas requiring further competency-based evaluations to support work redesigning that protects activities requiring registered nursing expertise, redistributes appropriate tasks, and reduces avoidable organisational inefficiencies.

## 1. Introduction

The global nursing workforce is facing unprecedented pressure due to persistent shortages, increasing healthcare demand, population ageing, and growing patient complexity [1]. In Europe and Italy, these challenges are reflected in increasing workload pressures [2,3], workforce instability [4,5], and caring for patients with increasingly complex needs [6]. Recent international reports indicate that workforce sustainability cannot be addressed solely through increasing the number of registered nurses, but requires strategies aimed at retaining professionals, improving working conditions, and ensuring that nursing competencies are effectively deployed within healthcare organizations [1,7,8].

Contemporary workforce challenges therefore require moving beyond staffing ratios alone, as numerical indicators do not fully capture how nursing resources are used in clinical practice. How nurses allocate their working time across may influence their ability to provide safe, timely, and effective care [7]. Inadequate allocation of nursing time may result from excessive workload, increasing administrative demands, interruptions, or the performance of activities that do not require registered nursing expertise, potentially limiting time available for clinical judgement, surveillance, and patient interaction [9]. Therefore, understanding how registered nurses spend their working time is essential to complement traditional staffing measures and to identify opportunities for workforce optimization, skill-mix redesign, and sustainable models of care delivery. Emerging approaches, including objective workload measurement systems, have highlighted the importance of assessing not only how many nurses are available but also how nursing time is allocated [10] and whether nurses are able to devote their time to activities that reflect their full professional scope of practice.

**Literature Review**

Observational studies show that nursing work is distributed across a wide range of activities: direct patient care, indirect care, medication management, documentation, communication, coordination, unit-related activities, personal or non-clinical tasks [9,11]. Recent time-and-motion studies have demonstrated that the distribution of nurses’ working time varies substantially across healthcare settings. In a large time-and-motion study involving 767 nurses across 36 medical–surgical units in USA, substantial proportions of nursing time were dedicated to documentation (35.3%), care coordination (20.6%), and medication-related activities (17.2%), while direct patient care represented a smaller proportion of total work time (19.3%) [9]. Similarly, a Swiss observational study conducted in an internal medicine unit observing 507.5 h of nursing practice, showed that registered nurses spent less than one-third of their working time in direct patient interaction, with communication, coordination, and care planning representing relevant components of daily practice and workload [11]. Conversely, some studies conducted in different acute care contexts in Turkey and UK have reported higher proportions of direct care time, highlighting the influence of clinical setting, activity classification systems, and methodological approaches on estimates of nursing work distribution [12,13]. Collectively, these findings indicate that nursing time cannot be interpreted solely through the proportion of direct care, as indirect care and other activities are integral components of safe and coordinated care delivery.

Beyond the distribution of activities, contemporary nursing work is characterized by competing demands, task switching, and interruptions that may increase cognitive workload and affect workflow continuity [14]. Time-and-motion research in South Korea has shown that nurses commonly experience interruptions during activities requiring sustained attention, including medication administration, documentation, and communication processes [14]. These workflow disruptions may increase cognitive demands, reduce continuity of tasks, and create additional challenges for maintaining patient safety [14]. Therefore, understanding nursing time allocation requires consideration not only of the duration of individual activities, but also of their nature, complexity, the competencies required, and the organizational conditions in which care is delivered. Differences observed across time and motion studies should be interpreted considering contextual factors such as patient characteristics, staffing levels, skill mix, ward organization, observation methods, and activity classification systems.

Beyond the distribution of nursing activities, an important consideration is whether the tasks performed by registered nurses are aligned with their professional competencies and responsibilities. Nursing work time cannot be interpreted solely according to whether activities occur at the bedside or outside direct patient contact, as different activities require different levels of expertise, clinical judgement, and accountability. The concept of illegitimate tasks provides a useful perspective for understanding activities that may conflict with professional role expectations, and includes two dimensions: unnecessary tasks, which could reasonably be avoided, and unreasonable tasks, which exceed the expected professional role or expertise [15,16,17]. In nursing contexts, examples may include administrative, logistical, or environmental activities that consume registered nurses’ time without requiring nursing-specific competencies [17]. However, these activities should not automatically be considered inappropriate or non-value-adding, as some may be necessary for effective care delivery. Recent multicentre evidence involving 1458 nurses in China showed that perceived illegitimate tasks were negatively associated with work engagement, highlighting the potential impact of inappropriate task allocation on nurses’ work experience [18].

An emerging issue in contemporary healthcare systems is therefore how to ensure that registered nurses’ time is directed toward activities requiring professional nursing expertise while maintaining effective care delivery through appropriate task allocation and skill-mix strategies. Increasing care complexity and workforce pressures have intensified the need to understand which activities require registered nursing competencies and which may be redesigned, delegated, or redistributed within multidisciplinary teams. Evidence on nursing time allocation so far remains heterogeneous. Variability in methodology regards sampling strategies, observer training, reliability assessment, and definitions of nursing activities [19]. Therefore, these differences limit direct comparisons between studies. Further research is needed to provide strong empirical evidence on how registered nurses’ working time is distributed in different healthcare contexts.

**Conceptual Framework**

A broader perspective is needed when interpreting nursing time. Nursing time allocation requires consideration not only of the duration and frequency of activities, but also on the nature of those activities, the competencies they require, and their alignment with organizational context and with nurses’ expected roles [20].

This study is anchored in the nursing scope of practice framework as its primary conceptual foundation, supplemented by Role Clarity [21] and Job Demands–Resources (JD-R) Theory [22] as associated explanatory lenses. Together, these frameworks provide a multi-tiered model for interpreting how organizational and environmental factors shape professional nursing practice and work time.

The Enacted Scope of Practice model refers to “the professional activities actually carried out by nurses, as opposed to the range of activities for which nurses are educated and licensed, and as distinct from the job responsibilities that might be expected on the basis of nurses’ qualifications and training” [20]. The model highlights the potential gap between nurses’ available competencies and their actual use in clinical practice, recognizing that scope enactment is influenced by both individual factors (e.g., education, experience, and professional development) and organizational factors (e.g., autonomy, workload, role expectations, and practice environment) [20,23]. The Actual Scope of Nursing Practice (ASCOP) operationalizes this concept across six domains: assessment and care planning, patient and family education, communication and care coordination, integration and supervision of staff, quality and patient safety, and knowledge updating and utilization [24]. ASCOP facilitates interpretation of registered nursing time devoted to activities requiring nursing-specific knowledge, clinical judgement, and professional accountability. From this perspective, a restricted enacted scope may occur when nurses’ competencies are not fully utilized, for example when a substantial proportion of their working time is allocated to activities that could potentially be redistributed within the healthcare team, limiting opportunities to engage in activities that require nursing expertise, such as patient education, quality improvement, and knowledge development [11]. Conversely, enabling nurses to practice closer to their full scope has been associated with improved patient care, organizational performance, and nurses’ satisfaction and well-being [25]. The extent to which nurses can enact their full scope of practice is also influenced by skill mix and task allocation within healthcare teams. Registered nurses and nursing support workers contribute differently to care delivery, performing distinct combinations of direct and indirect activities according to their competencies and responsibilities. Roche et al. [26] demonstrated substantial differences between regulated and unregulated nursing staff in the type of activities performed and in the occurrence of delayed or omitted care. These findings support the principle that workforce planning should consider not only the number of available professionals but also how activities are distributed according to competence, accountability, and required skill level.

Role Theory, initially developed within sociology and further expanded in organizational contexts by Kahn et al., ref. [21] provides an additional perspective for understanding how professional expectations and organizational demands shape nurses’ work activities. The theory proposes that individuals occupy social and professional roles characterized by expectations from multiple stakeholders, and that difficulties may arise when these expectations are unclear, incompatible, or excessive. Key concepts include role ambiguity (uncertainty regarding responsibilities and expectations), role conflict (competing demands arising from different expectations), and role overload (the perception that available time and resources are insufficient to meet all required responsibilities). In nursing, these concepts are particularly relevant because registered nurses operate at the intersection of clinical responsibilities, coordination requirements, administrative demands, and organizational expectations. When operational boundaries are ill-defined, nurses face role ambiguity and scope erosion, for example when engaging in clerical, administrative, or housekeeping functions that divert focus from core clinical responsibilities [27]. Therefore, nursing time allocation may also reflect the negotiation of multiple and sometimes competing roles within healthcare organizations. From this perspective, time spent on activities outside core nursing expertise may indicate organizational role expansion or role conflict rather than simply individual inefficiency. While the Enacted Scope of Practice model identifies whether nursing expertise is effectively utilized in practice, Role Theory provides insight into the organizational mechanisms that may restrict or expand this scope.

The enactment of nursing scope does not occur independently from the organizational environment. The Job Demands–Resources (JD-R) theory provides a complementary interpretive perspective by explaining how work characteristics influence the way nurses allocate their time [22]. According to this theory, job demands—including workload, time pressure, interruptions, administrative burden, and competing responsibilities—consume professional resources and may limit nurses’ ability to engage in high-value clinical activities [28]. Conversely, job resources, such as adequate staffing, teamwork, leadership support, autonomy, and effective organizational systems, may facilitate the use of nursing competencies and improve work processes [29]. Therefore, differences in nursing time allocation across settings may reflect not only patient needs but also the balance between organizational demands and available resources. While scope of practice and role clarity establish *what* nurses ought to do and *how* expectations are defined, JD-R Theory explains *how* the surrounding practice environment enables or hinders adherence to that scope. In resource-scarce environments, excessive job demands heighten role confusion and force nurses to spend time mitigating systemic inefficiencies [28]. Conversely, robust job resources protect professional boundaries, enhance role clarity, and buffer against burnout, allowing nurses to maintain their intended scope of clinical practice across diverse organizational settings [29].

**Problem Statement**

In Italy, nursing care is predominantly provided by registered nurses, who are regulated health professionals responsible for nursing assessment, care planning, implementation, evaluation, patient education, and coordination of care [30,31]. The professional autonomy and accountability of nurses are defined by national legislation and professional regulations [32,33]. Nursing assistants and other support workers, such as the *Operatore Socio-Sanitario* (OSS), may contribute to basic care activities and patient support within their defined competencies and in collaboration with nurses [34]. However, the organization of nursing work, skill mix arrangements, and distribution of activities between professional nurses and support personnel may vary across healthcare organizations and regions due to the decentralized organization of the Italian National Health Service [35]. Consequently, the allocation of nursing activities may differ according to local care models, workforce availability, and organizational strategies [35].

In Italy, nursing shortages are influenced by multiple factors, including historically low nurse-to-population ratios compared with other European countries [36], limited growth in nursing education capacity with nursing students reduced enrollment and withdrawing from their education programs [37], difficulties in recruitment and retention, an ageing workforce, and increasing turnover and retirement rates [38]. As a result, healthcare organizations face increasing pressure to maintain safe and effective care while optimizing the use of available registered nursing capacity.

Beyond workforce numbers, an important challenge concerns how registered nurses’ working time is allocated in clinical practice. In Italy, evidence on the actual distribution of registered nurses’ working time remains limited. Previous Italian studies have substantially contributed to understanding nurse staffing, missed nursing care [6,30,39], workload [2,3], and organizational factors influencing care delivery [40]. However, these studies relied mainly on self-reported measures and did not directly observe how nurses allocate their working time across different activities. More recently, Conti et al. [41] explored activities perceived as delegable among acute-care nurses in five Italian hospitals, providing important insights into potential task redistribution; however, findings were based on nurses’ perceptions rather than direct observation of nursing work processes. Consequently, current evidence remains insufficient to fully characterize how registered nurses spend their working time in contemporary hospital settings.

Expanding to international context, despite the growing literature on nursing time distribution, important knowledge gaps remain. Existing studies have described how nurses allocate their time across direct care, indirect care, documentation, communication, and organizational activities; however, most evidence was generated before or during the COVID-19 pandemic. Changes in healthcare delivery, increasing patient complexity, digital transformation, and evolving skill-mix models may have substantially reshaped contemporary nursing work [10,42]. Moreover, previous research has primarily quantified time spent on different activities, with limited attention to whether these activities require registered nursing expertise, reflect nurses’ enacted scope of practice, or represent opportunities for safe work redesign. Understanding the distribution of nursing time is therefore essential to describe how registered nursing competencies are currently utilized within healthcare settings.

**Purpose of the Inquiry**

The primary purpose of this multicentre observational study was to describe the distribution of registered nurses’ working time in medical–surgical hospital units in Italy.

Specifically, the study aimed to describe distribution, frequency, and duration of registered nurses’ observed activities during weekday morning and afternoon shifts in participating medical and surgical units and examine differences across participating hospitals and medical–surgical units.

A secondary purpose was to interpret the observed patterns of nursing activities through the lens of the Enacted Scope of Practice model, exploring how nursing time allocation relates to the use of registered nursing expertise, clinical judgement, and professional accountability. This interpretive approach aimed to identify areas that may warrant further investigation from a workforce organization and care redesign perspective.

The research question guiding this study is: “How is registered nursing time distributed across activities performed during weekday morning and afternoon shifts in medical–surgical hospital units in Italy and how does this distribution vary across hospitals and clinical units?”

**Originality, Rationale, and Significance**

The originality of this study lies in providing contemporary empirical evidence on registered nurses’ time allocation through direct multicentre observation of nursing activities in Italian medical–surgical hospital units. Unlike studies relying on staffing indicators, self-reported workload measures, or perceived task allocation, this study simultaneously examines the proportion of time, frequency, and duration of observed nursing activities during clinical shifts. Furthermore, the study integrates time-and-activity analysis with the Enacted Scope of Practice framework as an interpretive perspective to explore how registered nursing expertise is utilized in everyday clinical practice.

The rationale for this study derives from the limited availability of objective evidence describing what registered nurses in Italy actually do during their working shifts. Although staffing ratios, workload assessments, and nurses’ perceptions provide important information on workforce challenges, they cannot determine how nursing time is concretely distributed across different activities or whether available nursing expertise is fully utilized. Direct observation provides a complementary approach to identify the nature and organization of nursing work, generating evidence that may inform discussions on workforce organization, skill-mix models, and care delivery redesign.

The lack of objective evidence on how registered nurses allocate their working time limits the ability of healthcare organizations and policymakers to make informed decisions regarding workforce planning, skill-mix optimization, and care model redesign. By identifying how nursing time is distributed across direct care, indirect care, organizational activities, and other work processes, this study may support future strategies aimed at optimizing the use of nursing competencies while preserving registered nursing time for activities requiring clinical judgement, professional accountability, and specialized expertise. The findings may inform future investigations and organizational initiatives related to task allocation, delegation, and workforce redesign.

## 2. Materials and Methods

### 2.1. Design

This research adopted a descriptive observational multicentre design. The manuscript was prepared in accordance with the STROBE (Strengthening the Reporting of Observational Studies in Epidemiology) reporting guidelines for observational studies [43] (Supplementary File SC).

The Enacted Scope of Practice model [20], Role Theory [21] and JD-R [22] were adopted as the conceptual frameworks. In this study, these frameworks were not applied to classify individual activities or assign activities to specific result domains. Instead, the frameworks informed the interpretation of results by supporting the distinction between activities requiring registered nursing expertise, clinical judgement, and professional accountability, and activities that may represent opportunities for redistribution or redesign within the healthcare team.

### 2.2. Setting and Participants

This multicentre study was conducted in five teaching hospitals located in northern, central, and southern Italy, including both public and private healthcare institutions. Hospitals were recruited through the Italian Association of Nurse Directors (SIDMI). Following an invitation distributed through the association, nurse directors voluntarily expressed their interest in participating in the study. Only the five hospitals included in the study accepted participation.

After local ethics approval of the study, each participating hospital identified medical–surgical units eligible for inclusion. Unit selection was performed purposively by the hospital nurse directors according to predefined criteria: adult medical–surgical wards with routine inpatient activity, nursing staff willing to participate, and feasibility of conducting observations. Each hospital contributed more than one unit, resulting in the inclusion of thirteen medical–surgical wards. Specialized clinical areas, including intensive care units, sub-intensive care units, paediatric units, emergency departments, and other highly specialized settings, were excluded because of differences in workflow organization and activity patterns.

Data collection was conducted in each unit over three consecutive weeks, about fifteen observed shifts, between 2021 and 2024.

Observation periods were identified before data collection in collaboration with nurse managers to capture routine clinical activity. Atypical periods were excluded when characterized by planned reductions in activity, holiday periods, increased staff absenteeism, or unusual organizational conditions. Although this approach aimed to ensure comparability across observations, the identification of typical periods involved managerial judgement and did not represent random temporal sampling.

Within each observation period, morning and afternoon shifts were selected using a computer-generated randomization procedure among eligible shifts. However, in some cases, the initially selected shift had to be rescheduled because of unexpected observer unavailability or because nurses were not available/willing to participate during that specific shift. Therefore, while random selection was used as the initial strategy, the final observation schedule was influenced by operational feasibility.

Eligible participants were registered nurses providing direct patient care in the participating units. Inclusion criteria were: full-time employment, routine assignment to the selected unit, and at least two months of experience in the ward. Nurses working as float staff, performing overtime or additional shifts, or not routinely assigned to the unit were excluded. In total, 268 nurses across the 13 included units met the eligibility criteria. Of these, 209 participated in the study, while 59 declined.

During each shift, one registered nurse was observed for the entire duration of the shift. Nurse participation was voluntary, and different nurses were selected whenever possible to maximize variability across observations. Whenever possible, different nurses were selected for each observation. When this was not feasible due to staff availability, repeated observations of the same nurse could occur. This was adopted to maintain the planned observation schedule and avoid concentrating observations on fewer available staff members. Nurse identifiers were not collected; therefore, repeated observations could not be completely excluded.

### 2.3. Data Collection System

The monitoring system was supported by a purpose-built mobile application designed to facilitate and standardize the observation process conducted by trained human observers. The application was developed to address the need for systematic, real-time data collection of nursing activities while minimizing observer burden and ensuring data consistency across multiple observation sessions. The mobile application operated entirely on the local device, eliminating the need for continuous network connectivity during data collection periods and ensuring data security.

The application implemented a hierarchical, tree-based interface structure that enabled rapid identification and selection of nursing tasks from a comprehensive taxonomy.

The taxonomy of nursing activities was developed by the research team, including two expert nurses with more than 10 years of clinical experience in medical and surgical units. The development process was informed by previous literature on nursing time-and-motion studies and observational approaches describing nursing work processes. An initial list of activities was generated to capture the main components of nursing work. The preliminary taxonomy was reviewed by the expert nurses to assess the clinical relevance, completeness, and clarity of activity definitions. Subsequently, the taxonomy was pilot-tested during four complete nursing shift observations conducted in medical–surgical units before the application development. During this phase, observers applied the taxonomy in real clinical settings to evaluate the feasibility of activity classification and identify potential gaps. Activities observed during pilot shifts that were not represented in the initial taxonomy were integrated, and activity definitions were refined accordingly. Following this refinement process, activities were organized into a hierarchical structure, allowing observers to navigate efficiently through multiple levels of task categories, from broad classifications (e.g., direct care, indirect care, and personal time) to specific activities (e.g., medication administration, patient assessment, and documentation). The final taxonomy, including all activity categories, individual activity codes, and classification definitions, is reported in Supplementary File SA.

The hierarchical organization was implemented within the mobile application used for data collection. This structure allowed observers to select the appropriate activity during real-time observation, maintain a standardized classification system across units and hospitals, reduce selection time and potential classification errors. For each observed activity, the application automatically recorded temporal information, including start time, end time, and duration.

Observers initiated task recording by selecting the appropriate activity from the hierarchical menu, and the application automatically timestamped the event. Upon completion of the activity, observers closed the recording, and the application calculated the elapsed time. This functionality provided precise measurement of nursing work temporal patterns throughout the observation period.

The application also allowed observers to record simultaneous activities, reflecting the multitasking nature of nursing practice. When two activities occurred concurrently, both activities could be recorded with their respective timestamps, without interrupting the ongoing recording. For example, while a nurse was measuring a patient’s temperature and oxygen saturation, the observer could record the clinical assessment activity and simultaneously code documentation or communication activities if these occurred concurrently. Similarly, if an interruption occurred during an ongoing activity, the interruption could be recorded as a separate event while maintaining the original activity record. Therefore, recorded activity durations could overlap, and the cumulative duration of activities could exceed the chronological observation time. Results were consequently expressed as proportions of recorded activity time.

All recorded events were stored in a local database on the mobile device, ensuring data integrity and preventing loss due to connectivity issues. The database structure was designed to capture essential information including task identification codes, timestamps, duration, and observer identifiers. This local storage approach also facilitated offline data collection in clinical environments where network access might be restricted or unreliable. Finally, the application incorporated a data extraction functionality that enabled export of collected data in a standardized format suitable for subsequent statistical analyses. This feature allowed researchers to retrieve complete datasets from multiple devices and consolidate them for analysis while maintaining data structure and integrity.

### 2.4. Data Collection Procedure

Data collection was conducted across five hospitals over a period of approximately 40 months, from February 2021 to June 2024. The data collection process was not continuous but occurred during specific observation periods when access to participating units was granted and trained observers were available. Within the five participating hospitals, a total of 13 nursing units were included in the study. The distribution across hospitals was as follows: Hospital 1 (n = 5 units), Hospital 2 (n = 1 unit), Hospital 3 (n = 4 units), Hospital 4 (n = 1 unit), and Hospital 5 (n = 2 units). All participating units were either general medicine or surgery departments, representing two of the most common inpatient care settings where nursing work characteristics could be systematically compared.

In each participating unit, observations were conducted over three consecutive weeks, with observations taking place five days a week, from Monday to Friday. About 15 shifts were observed in each unit including morning and afternoon shifts. Weekends and night shifts were excluded from the observations, as the nursing work and activities during these times can differ from those on regular weekday shifts. Observations were conducted during morning and afternoon shifts to obtain a comprehensive representation of nursing work patterns. Morning observation sessions typically spanned from 7 AM and 2 PM, while for afternoon shifts, observations were conducted from 2 PM to 9 PM. This approach allowed for the documentation of work variations across different shift periods and times of day.

All observations were performed by trained observers using the mobile application described above. The researchers organized an 8-h training session for observations at each participating institution. The first 4 h focused on explaining the shadowing observation methods, identifying the types of nursing activities to be observed and recorded, and training on the use of the mobile application. The remaining 4 h were dedicated to field observations, where observers recorded real-time nursing activities in the unit. During the training phase, selected observations were conducted in parallel by the trained observer and a researcher to assess the consistency of activity identification and classification. Both observers independently recorded nursing activities using the same taxonomy and mobile application procedure. Any discrepancies were discussed to clarify activity definitions and improve observer understanding. Specific procedures for recording concurrent activities, multitasking situations, interruptions, and the appropriate selection of activity codes were discussed and clarified with the trainer. Particular attention was given to ensuring that observers correctly identified the primary activity and any concurrent activities performed by nurses during complex workflow situations. All observers were trained by the same researcher to ensure consistency in the data collection methodology. The researcher periodically supervised observation procedures during data collection and discussed potential classification uncertainties with observers to ensure consistent application of the taxonomy.

Shadowing is a data collection technique that involves observing participants within their natural settings to gain insight into their actual behaviors, activities, and contextual interactions [44]. The methodology entails the researcher shadowing the participant for a specific time, aiming to minimize disruption, preserve the participant’s natural behavior, and capture the full depth of their experiences. The researcher’s role is purely observational, with no interaction with the participants. The observers were either final-year bachelor nursing students or registered nurses working in different units than the ones being observed. To accurately capture each activity performed by nurses during their shift, it was crucial that the observers were both competent and confident in nursing practices. Therefore, the selection of observers was strategic, ensuring they could recognize the activities being carried out and know which actions to document in the application. Based on the availability of observers, each observer was assigned to a single unit, and whenever possible, the same observer was dedicated to a specific institution throughout the study. This approach was used to reduce data collection bias and ensure uniformity in the data collection process.

Observers maintained continuous recording of nursing activities throughout each observation session (full shift length), capturing the full range of direct care, indirect care, personal time or other activities. For morning shift observations, the typical observation period was between 7 AM and 2 PM, while for afternoon shifts, observations were conducted from 2 PM to 9 PM. Following each observation period, exported records were combined and reviewed by the authors for correctness and consistency. Records reporting the same activity less than one second apart were treated as likely accidental double entries by the observer and removed. The proportion removed was estimated at 1–2% of exported records; an exact count is not available. Activity categories assigned by observers during recording were retained without retrospective reclassification. No other errors requiring correction or exclusion were identified. Derived quantities were calculated from the recorded data; no missing values were imputed and no new observations were added. The original exports were preserved.

Recorded nursing activities were systematically categorized into four activity categories according to their nature and function within the nursing workflow. Operational definitions and areas of activities are described in Table 1.

See Supplementary File SA for additional details. This categorization was applied consistently across all observation sessions and hospitals, enabling standardized comparison of nursing work composition across different units and settings.

### 2.5. Data Analysis

Data preparation, including review of exported records and removal of likely duplicate entries, is described in Section 2.4. To enable quantitative comparison of nursing work patterns across units and hospitals, several metrics were derived from the categorized activity data. The primary metric was unit time, defined as the mean duration per recorded activity event within each category. It corresponds to the total recorded duration in a category divided by the number of contributing events. Equivalently, it is the average of the mean durations of the constituent activity types, weighted by their recorded frequencies.

Formally, for each activity category *c*, the unit time metric *T_c_* of this category was computed as:

$$T_{c}=\frac{\sum_{a\in A_{c}}n_{a}T_{a}}{\sum_{a\in A_{c}}n_{a}}$$

where T*a* represents the arithmetic mean duration in seconds of recorded events of activity type *a* belonging to category *c*, n*_a_* is the number of events of that type contributing to the calculation and A*_c_* is the set of observed activity types belonging to category *c*. The numerator therefore represents cumulative activity duration and the denominator the number of contributing events in the category; unit time is expressed in seconds. This mean value summarizes recorded time per event but does not describe the spread or shape of the duration distribution This metric serves a dual interpretive purpose. First, it represents the mean duration per recorded activity event, providing insight into how much time nurses typically allocate on average to individual tasks within each category. Higher unit time values indicate longer average recorded durations but do not by themselves establish greater task complexity or thoroughness of execution. Second, from a workforce planning perspective, this metric can be interpreted as the time cost of a single activity unit within each category. Activity duration and frequency can inform workload planning, but cannot alone determine required staffing levels.

Beyond the unit time metric, additional descriptive measures were computed to characterize the overall organization of nursing work across the observation periods. The time fraction for each activity category was calculated as the proportion of total recorded activity time allocated to each of the four activity categories. For the overall sample or the specified stratum, this metric was defined as:

$$f_{c}=\frac{\sum_{a\in A_{c}}n_{a}T_{a}}{\sum_{c}\sum_{a\in A_{c}}n_{a}T_{a}}$$

where f*_c_* represents time fraction for category *c*. This metric provides insight into how the recorded activity is distributed across direct care, indirect care, organizational activities, and non-patient care time. The time fraction metric enables comparison of work composition patterns across different units, hospitals, and shifts, revealing whether certain settings require proportionally more time dedicated to specific activity types.

Additionally, the category frequency distribution was computed to quantify how often nurses engaged in different types of activity categories regardless of duration. This was expressed as the percentage of total recorded activities falling within each category:

$$p_{c}=\frac{\sum_{a\in A_{c}}n_{a}}{\sum_{c}\sum_{a\in A_{c}}n_{a}}\times 100$$

This metric allows distinguishing between time-intensive but infrequent activities and brief but frequently repeated tasks, both of which contribute differently to overall nursing workload.

All statistical analyses were performed using R. Descriptive statistics were computed for all work metrics: for unit time per activity category (T*_c_*), time fractions (f*_c_*), and category frequency distributions (p*_c_*). These descriptive measures were calculated both for the overall dataset and stratified by hospital, and department type (medicine vs. surgery).

Global time percentages were calculated as the cumulative duration of each category divided by the cumulative duration of all activities included in the analysis, expressed as a percentage. Durations were pooled across observation sessions; session-specific percentages were not averaged. The denominator was total recorded activity time, including overlapping activities, rather than elapsed shift time. No additional weighting was applied to give nurses, units, or hospitals equal contributions. Their contributions to global time percentages were proportional to recorded activity duration; hospitals contributing more units could therefore contribute more through a larger cumulative duration. Activity frequency percentages were calculated as the number of events in each category divided by the total number of activity records included in the corresponding analysis, expressed as a percentage. These summaries describe the observed sample and do not represent an equally weighted average across hospitals.

The activity event is the record-level unit summarized in these descriptive analyses. Activity events are nested within observation sessions, each corresponding to one nurse observed during one shift; sessions are nested within units, and units within hospitals. Repeated observations of individual nurses may introduce additional dependence. As nurse identifiers were not collected, the contribution of repeated observations of the same nurse cannot be assessed. Hospital- and department-specific descriptive summaries do not constitute an adjustment for clustering. Cluster-adjusted measures of uncertainty are not reported, and comparisons should be interpreted as descriptions of the observed sample.

To provide a more detailed description of the recorded nursing activities within the four overarching categories, a further descriptive analysis of the original activity records was conducted for direct care, indirect care, organizational activities, and non-patient care. All records assigned to each overarching category were extracted and grouped into predefined subcategories, as described in Supplementary File SA. For each subcategory, the number of activity records and cumulative duration were calculated. The proportion of activity records for each subcategory was calculated by dividing the number of records in that subcategory by the total number of records within the corresponding overarching category. Similarly, the proportion of recorded time was calculated by dividing the cumulative duration of each subcategory by the total recorded duration of its corresponding overarching category. All percentages reported for the subcategories represent the internal composition of the relevant overarching category and do not represent proportions of total observed nursing time.

### 2.6. Potential Sources of Bias

Several potential sources of bias were considered and addressed during study design and data collection.

Selection bias: To reduce the possibility that findings reflected the workflow of a single organization, nurse or clinical specialty, observations were conducted across multiple hospitals, including both medical and surgical units from different geographical areas. Within each setting, observations covered complete nursing shifts rather than selected periods of activity.

Temporal sampling bias: Nursing work and activity patterns may vary according to the time of day and day-to-day clinical circumstances. To improve temporal representativeness, data collection was conducted over a three-week observation period and included both morning and afternoon shifts.

Observer bias: Because activities were recorded through direct observation, classification and recording could be influenced by the observer. To limit this source of bias, observers followed a specific training, used standardized observation procedure and a predefined classification of nursing activities and operational definitions for recording activities.

Measurement/recording bias: Differences in observer attention or accuracy could affect the identification, timing, or recording of activities, particularly brief, simultaneous, or frequently interrupted activities. The use of predefined activity categories and a standardized data-collection approach was intended to improve consistency in recording.

Misclassification bias: To reduce inconsistent attribution of observed activities, nursing work was prospectively organized into predefined categories—direct care, indirect care, organizational activities, and non-patient care activities—and the same classification was applied across settings and observations.

### 2.7. Ethical Considerations

The study was approved by the five local Ethics Committees (Prot.: 95/20 OSS ComEt CBM, 27 October 2020; Verbale N. 62; 14 May 2021; Prot N. 0116717/2021, 13 November 2021; Prot N IRRB 13/22, 21 September 2022; Prot N.58/2022, 11 October 2022). The researchers approached the nurses in each center individually, explaining the aims of the study. After reading the written information, nurses signed the informed consent. Those who refused to sign the informed consent were excluded from the study. Data access was restricted to the research team. The study was conducted in accordance with the standards and recommendations of the Declaration of Helsinki and the European Union General Data Protection Regulation (GDPR) guidelines.

## 3. Results

### 3.1. General Data Description

Between February 2021 and June 2024, observations were conducted in 13 medical–surgical units across five teaching hospitals in northern, central, and southern Italy. In Hospital 1 were observed 65 shifts in 5 units, 2 medicine and 3 surgery departments. In Hospital 2 were observed 15 shifts in a medical unit. In Hospital 3 were observed 56 shifts in 2 medicine and 2 surgery units. In Hospital 4 were observed 13 shifts in a surgical unit. In Hospital 5 were observed 28 shifts in 1 medical and 1 surgical unit. The exact number of unique nurses observed could not be determined because nurse identifiers were not collected. Overall 177 nursing work complete shifts were observed: 96 mornings and 81 afternoons. A total of 161,094 h of observation were performed and 43,900 nursing activities have been recorded.

### 3.2. Distribution, Duration, and Frequency of Nursing Activities

The distribution of recorded activity time differed across the four predefined activity categories (see Table 2).

At the global level, direct care accounted for 45% of total recorded activity time, followed by indirect care (24%), non-patient care (21%), and organizational activities (10%) (Table 2A). Thus, almost half of the recorded activity time was allocated to activities performed directly with patients, whereas the remaining 55% was distributed across indirect, organizational, and non-patient care activities.

Time allocation varied across hospitals (Table 2B). Direct care showed the lowest between-hospital variability, ranging from 42% to 48% of total recorded activity time. Greater variability was observed for indirect care (19–40%), organizational activities (5–18%), and non-patient care (10–28%). Hospital 3 showed the highest overall proportion of direct care (48%), Hospital 4 the highest proportion of indirect care (40%), Hospital 2 the highest proportion of organizational activities (18%), and Hospital 5 the highest proportion of non-patient care (28%). These findings suggest that differences between hospitals were driven primarily by the distribution of activities performed outside direct patient care rather than by major differences in the proportion of direct care itself.

Differences also were observed between surgical and medical departments (Table 2C). Direct care represented 51% of total recorded activity time in medical units compared with 41% in surgical units. Conversely, indirect care accounted for a larger proportion of recorded activity time in surgical than in medical units (28% vs. 17%). Organizational activities represented a similar proportion in the two settings (10% in surgical and 11% in medical units), while non-patient care accounted for 21% of recorded activity time in both. Organizational nursing activities represented a heterogeneous domain including quality and safety checks, administrative procedures, delegation and supervision activities, education and training, isolation management, environmental/logistical activities, and communication processes. Detailed distribution of the individual activities included in this category is reported in Supplementary File SB. The non-patient care category included different types of activities. These included personal time, as well as work-related periods such as movement within the ward, interruptions, waiting, and other workflow-related activities. Personal time represented only a limited proportion of recorded activity time compared with other components of this category. Hospital-level stratification showed additional heterogeneity. The largest contrast occurred in Hospital 3, where direct care accounted for 65% of recorded activity time in the medical unit but 35% in the surgical unit. Indirect care showed the opposite pattern in the same hospital, accounting for 8% and 41% of recorded activity time, respectively. A detailed classification of nursing work time distributed among hospitals and units on the four classes of activity is presented in Supplementary File SB.

Analysis of activity frequency provided complementary information on nursing workflow (Table 3). Direct care was also the most frequently recorded category, accounting for 48% of all discrete activities (Table 3A). Indirect care represented approximately one-quarter of recorded activities, while non-patient care accounted for approximately one-fifth. Organizational activities were the least frequent, representing only about 5–6% of all recorded activities.

Department-specific frequency patterns were consistent with the time-fraction analysis but were not identical (Table 3B). Direct care activities occurred more frequently in medical than in surgical departments, accounting for approximately 52% and 45% of recorded activities, respectively. In contrast, indirect care activities were somewhat more frequent in surgical units (approximately 26% vs. 22%). Organizational activities remained infrequent in both department types, while non-patient care activities represented approximately one-fifth of recorded activities. Hospital-level analysis further demonstrated heterogeneity in workflow composition. The medical unit of Hospital 3 showed the most pronounced predominance of direct care activities (Table 3C), whereas other hospitals displayed a more balanced distribution among direct, indirect, organizational, and non-patient-care activities (Table 3D).

The analysis of unit time (mean activity duration) added a third dimension to these findings (Table 4). Organizational activities showed the longest mean duration, averaging 210 s globally, with a longer mean duration in medical than in surgical departments (255 vs. 190 s).

This pattern, differently from the low frequency, indicates that organizational activities occurred relatively infrequently but had the longest mean duration when performed.

Taken together, the three measures showed different but complementary dimensions of nursing work. Direct care displayed relatively close similarities between its share of activity frequency and total recorded activity time (48% of activities and 45% of time). A similar correspondence was observed for indirect care, which represented approximately one-quarter of both recorded activities and total recorded activity time, and for non-patient care, which accounted for approximately one-fifth of both measures. The largest variation was observed for organizational activities. Although they represented only approximately 5–6% of recorded activities, they accounted for 10% of total recorded activity time and had the longest mean activity duration. This finding supports that activity frequency alone does not fully capture differences in the duration and overall time allocation of nursing activities.

### 3.3. Description of Nursing Activities Within Classification Categories

#### 3.3.1. Direct Patient Care Activities

This category included 21,366 direct-care activity records corresponding to a cumulative duration of 760.62 h. Medication administration was the most frequently recorded direct-care subcategory, accounting for 34.37% of direct-care activity records and 25.65% of cumulative direct-care time. Vital signs monitoring represented 28.55% of recorded activities and 18.69% of direct-care time, while admission/discharge/patient communication accounted for 8.79% and 14.01%, respectively. Hygiene care represented 4.35% of direct-care activity records but 9.44% of cumulative direct-care time. The remaining subcategories each accounted for less than 6% of cumulative direct-care time. These percentages describe the internal composition of the direct-care category and do not represent proportions of total observed nursing time. The distribution of direct care subcategories according to activity frequency and cumulative duration is summarized in Table 5.

#### 3.3.2. Indirect Patient Care Activities

This category included 10,812 indirect-care activity records corresponding to a cumulative duration of 396.95 h. Medication preparation was the most frequently recorded indirect-care subcategory, accounting for 28.51% of activity records and 17.00% of cumulative indirect-care time. Medical record review/data entry represented 27.59% of recorded activities and 18.18% of indirect-care time. Communication, combining communication with other healthcare professionals, nurse managers, nursing colleagues, and physicians, accounted for 17.72% of activity records and represented the largest proportion of cumulative indirect-care time (23.21%). Nursing documentation/review accounted for 18.47% of activity records and 18.69% of indirect-care time. Notably, verbal patient handover represented 5.02% of recorded activities but 15.91% of cumulative time. Multidisciplinary ward rounds and dietary care planning accounted for smaller proportions of both activity frequency and cumulative duration. These percentages describe the internal composition of the indirect-care category and do not represent proportions of total observed nursing time. The distribution of indirect-care subcategories according to activity frequency and cumulative duration is summarized in Table 6.

#### 3.3.3. Organisational Activities

The organizational activity category comprised 2766 recorded activities, corresponding to a cumulative duration of 155.16 h. The distribution of subcategories showed a notable difference between the frequency of activities and the proportion of organizational time they occupied (Table 7). Delegation was the most frequently recorded subcategory, accounting for 32.00% of organizational activity records and 26.55% of organizational time. Managerial and organizational tasks accounted for the largest share of organizational time (37.65%) despite being recorded less frequently (21.26%). Training activities similarly occupied a greater proportion of organizational time (17.48%) relative to their frequency (9.69%). In contrast, telephone communications and isolation-related activities were recorded relatively frequently but accounted for comparatively smaller proportions of organizational time. Computer-related activities were rarely recorded and contributed minimally to the overall organizational time. Overall, the findings indicate that the frequency with which organizational activities occurred did not necessarily correspond to the amount of time they required, with some less frequent activities accounting for a disproportionate share of organizational time.

#### 3.3.4. Non-Patient Care Activities

The non-patient care included 8956 recorded activities, corresponding to a cumulative duration of 298.21 h. Grouping those activities within subcategories showed that ward movement accounted for the largest proportion of non-patient care time (42.78%), followed by interruptions (35.39%) and personal time (15.38%). Time outside the ward and waiting accounted for 3.98% and 2.47% of non-patient care time, respectively. In terms of activity-record frequency, ward movement represented 47.72% of non-patient care activity records and interruptions represented 45.24%. Personal time accounted for 5.14% of records, waiting for 1.05%, and time outside the ward for 0.85% (Table 8). These percentages describe the internal composition of the non-patient care category and do not represent proportions of total observed nursing time.

## 4. Discussion

The present study provides a contemporary multicentre description of how registered nurses allocate their working time across medical and surgical hospital units in Italy. The principal finding is that nursing work was distributed across multiple interconnected domains rather than being concentrated exclusively at the bedside. Overall, approximately 45% of recorded activity time was devoted to direct care and 24% to indirect care, indicating that almost seven out of every ten minutes of recorded activity time were associated with activities directly or indirectly supporting patient care. The remaining time was distributed across organisational and non-patient care activities, with substantial variation between units and hospitals.

**Distribution of registered nursing working time**

The 45% share of recorded activity time allocated to direct-care in the present study represents a substantial component of nursing work. These findings support a more favorable allocation of clinical time and a potentially stronger patient-care orientation in our settings. In the international literature, the proportion of time nurses allocate to direct patient care shows a remarkably consistent trend, a mean of 40% [11,42,45,46], despite differences in the countries healthcare models, ranging from universal public systems (e.g., UK, Finland, Sweden, Brazil, and Australia) to mixed systems (e.g., Jordan, Bangladesh, Turkey, and Switzerland) or predominantly private-insurance-based systems (e.g., United States). This suggests that the limitations in nursing time may not solely be a function of the healthcare funding model, but rather may reflect common organizational challenges linked to work environments, organization of work, workload, documentation requirements, or skill mix. The findings as well support that direct care accounts for less than half recorded activity time close to the patient and that direct bedside time alone is not an adequate indicator of the contribution of nursing work to patient outcomes.

Approximately 24% of recorded activity time in the present study was allocated to indirect nursing activities, although marked variability (19–40%) was observed across clinical contexts. These activities included documentation and record review, patient handover, communication with healthcare professionals, multidisciplinary ward rounds, care-related data management, and medication preparation. They frequently occur away from the bedside but are integral to continuity, coordination, medication safety, surveillance, and clinical decision-making. Therefore, a high proportion of indirect care time should not in itself be considered inefficient. Internationally, different proportions of indirect activity, from 12–68%, have been reported [11,42,47] suggesting the need for caution when making direct comparisons. Such heterogeneity likely reflects differences in ward organisation, patient complexity, staffing and skill mix, but also methodological differences in the definition and classification of nursing work [42]. In addition, this variability can mirror broader system-level transformations, especially digitalization. Electronic health records and other information systems can facilitate accessibility, continuity, and safety but may also increase documentation demands when systems are poorly integrated or when information must be entered repeatedly. Evidence from time-motion and EHR-focused studies shows that documentation can consume a substantial portion of nurses’ time in the shift (about 30%), reshaping the balance between direct and indirect care and often contributing to perceptions of workload and inefficiency [48,49]. Several authors argue that when indirect care becomes dominant or poorly supported, it can lead to suboptimal use of the nursing scope of practice, constrain relational time with patients and families, and ultimately lead to missed care, moral distress and burnout [11,50]. In this sense, in some work contexts in our study, high indirect activity data may be read as a signal that nurses in these settings are heavily engaged in “invisible” coordination and documentation work. Managers are suggested to critically examine which components of indirect care contribute to care quality and require registered nursing expertise, and which instead reflect system complexity, duplication, inefficient information systems, or avoidable administrative burden.

A further component of nursing work was represented by organisational activities, which accounted for approximately 5–18% of recorded activity time across settings. This range is fully consistent with previous evidence [45,46,47]. These activities included quality-control checks, administrative procedures, cleaning and restocking of clinical areas and medications, delegation and supervision, education and training, isolation-related organisation, and telephone communication with internal and external services. Some of these activities are clearly part of the registered nurse’s coordinating and safety role. Quality checks, supervision, clinical communication, and education, for example, may depend substantially on nursing competence and professional accountability. Others, however, raise questions about whether registered nursing expertise represents the most appropriate resource for their performance. The organizational activity category should be interpreted cautiously, as it includes both professional nursing activities requiring clinical responsibility and coordination, as well as operational tasks influenced by local organizational models. Therefore, the potential for redistribution should not be inferred from the overall proportion of organizational time but should be evaluated at the level of specific activities and competencies required.

Finally, approximately 21% of recorded activity time was classified within the overarching category non-patient care, with substantial variation across units. Non-patient care time should be interpreted cautiously, as it encompasses conceptually distinct activities. Personal breaks represent legitimate recovery time, whereas movement, interruptions, and waiting may reflect workflow characteristics, organizational constraints, or care coordination demands. Therefore, this category should not be considered synonymous with inefficient use of nursing time. In some international studies, non-patient care time was classified as only 10% [46,47] and only in one study in Turkey a 32% of time was reported [12]. Within this category, interruptions and movement are particularly relevant from an organisational perspective because they may reflect the physical and procedural design of work rather than individual nurse behaviour. The considerable variation observed between units in the present study similarly suggests that at least part of this time may be organizationally modifiable rather than intrinsic to nursing practice. Kramer et al. [42], for example, documented frequent interruptions during nursing work, every 12 min, most commonly occurring during documentation, but also during direct care (11%) and medication administration (9%). Previous studies have associated workflow interruption with increased cognitive demands and potential higher error risk and reduced quality of care [51,52]. The critical issue that may arise, therefore, is not simply the amount of time spent on non–patient care activities, but the possible lack of systemic safeguards to prevent unnecessary interruptions and reduce task fragmentation. The fact that only a minority of studies report high percentages suggests that these problems may arise from organizational choices, inadequate workflow design, and insufficient protection of nurses’ cognitive bandwidth. Future studies should further disaggregate the non-patient care components to better understand which elements represent necessary workflow processes and which may indicate opportunities for organizational improvement.

Observed variation between nursing activity time and hospitals in this study should be interpreted cautiously. Differences may reflect not only organizational characteristics but also the different calendar periods in which observations were conducted. Changes in staffing conditions, care processes, documentation systems, and organizational practices between 2021 and 2024 may have contributed to the observed variation. However, the findings of this study suggest that current organizational models may contribute to suboptimal allocation of nursing time, with potential implications for patient care. These findings highlight the need to critically examine whether current organizational models sufficiently support efficient workflows and enable registered nurses to focus on activities requiring their professional competencies. Previous research suggests that insufficiently structured delegation processes, frequent interruptions, and limited protection of interruption-free zones for high-risk activities may contribute to fragmented workflows [51,52]. Organizational interventions aimed at clarifying roles, strengthening structured delegation processes and improving workflow design may represent potential strategies to optimize nursing time allocation, ultimately improving both patient outcomes and nurses’ professional satisfaction [48]. The relevant question is therefore not simply how much time nurses spend with patients, but whether registered nursing time is being used for activities in which nursing knowledge, clinical judgement, and professional accountability generate the greatest value. Future studies should distinguish necessary clinical and organizational activities from avoidable workflow overhead and examine whether interventions addressing ward layout, supply availability, communication processes, or interruption management can improve the allocation of nursing time without restricting legitimate breaks or compromising care.

**Enacted Scope of Practice and the organisation of nursing work**

The distribution of nursing time observed in this study can be further interpreted through the Enacted Scope of Practice model [20]. The framework distinguishes the competencies and activities for which registered nurses are educated and authorised from the professional activities they are actually able to perform in everyday practice. In the present study, the framework provides an interpretive lens to consider whether the observed allocation of nursing time enables registered nurses to fully apply their professional competencies. Accordingly, the findings do not quantify the proportion of activities that were concordant with registered nursing scope, required nursing-specific expertise, or could be appropriately redistributed to other personnel.

From this perspective, activities such as comprehensive patient assessment, clinical surveillance, identification of deterioration, care planning and evaluation, complex medication management, therapeutic education, clinical coordination, communication with patients and families, and supervision of the care process represent areas in which registered nursing competence may provide distinctive clinical value. Many of these activities were captured within the direct and indirect care categories identified in this study. Consequently, protecting nurses’ enacted scope of practice does not mean maximizing direct care percentages indiscriminately; it means ensuring that registered nursing time is preferentially available for those components of care that depend on nursing-specific competence [20,25].

Conversely, the findings also highlight that registered nurses perform a broad range of organizational and workflow-related activities that could potentially be reconsidered. The organisational activity data are particularly relevant in this respect because nurses were observed performing both high-accountability activities, such as quality-control checks and supervision, and activities for which the added value of registered nursing expertise may be more limited. Evidence suggests that the enactment of nursing scope is influenced by organizational and work-related conditions, meaning that improving scope enactment may require changes to role design, staffing, workflow, and support systems rather than delegation alone [20,25]. Similarly, the substantial time represented by movement and interruptions suggests that some opportunities to improve scope enactment may depend on workflow and system redesign, including better coordination of supplies, information, communication, and task sequencing. The objective is not to maximise direct care time as an isolated metric, but to maximise the proportion of nursing time devoted to activities that require registered nursing competence and contribute directly or indirectly to safe, coordinated, and evidence-informed care. The present study did not apply a formal task-level classification of competency requirements, appropriateness, or delegability. Consequently, decisions about workforce redesign should be made at the level of individual tasks rather than the aggregated categories. Such decisions would require explicit assessment of clinical purpose, contextual necessity, required competence, professional accountability, applicable regulation, and the availability and preparation of other personnel.

The interpretation of these findings can be further supported by Role Theory [21]. The distribution of nursing time observed across the four activity domains identified in this study reflects the multiple and sometimes competing expectations embedded in contemporary nursing roles. From a Role Theory perspective, the coexistence of multiple expectations does not necessarily indicate inefficient use of nursing time. Rather, it may reflect the inherently multifaceted nature of the registered nurse role within modern healthcare organizations. Contemporary nursing work encompasses not only direct clinical care but also care coordination, management of the care environment, patient education, quality improvement, and activities that enable the functioning of the wider healthcare system [53,54]. Nurses may therefore distribute their time across several interconnected functions because these activities collectively support continuity, safety, and organizational capacity. Nevertheless, role complexity should be distinguished from role ambiguity and role overload. When expectations are unclear, incompatible, or inadequately supported, the coexistence of roles may generate stress, burnout, and difficulties in maintaining continuity of care [55]. This perspective supports future organizational evaluations aimed at clarifying professional responsibilities, assessing task allocation according to competency requirements, and identifying whether workflow redesign may better align nursing activities with expected professional roles.

The Job Demands–Resources (JD-R) theory [22] provides an additional perspective for interpreting the study findings. The observed distribution of nursing time across direct care, indirect care, organizational activities, and non-patient care domains reflects the relationship between the demands embedded in nursing work and the resources available to manage them. Documentation, communication, and care coordination may be necessary for safe and continuous care; however, they may also function as additional demands when they occur alongside high workload, staffing shortages, time pressure, and fragmented work processes [29,56]. Conversely, adequate staffing, teamwork, autonomy, role clarity, and supportive leadership may operate as job resources because they help nurses achieve work goals, reduce the costs associated with demanding tasks, and preserve time for activities requiring clinical judgment and patient interaction [57]. From this perspective, future workforce redesign initiatives should not focus exclusively on reallocating tasks; they should also strengthen the organizational and relational resources that enable nurses to practice effectively and sustain work engagement.

**Implications for practice**

The findings support a cautious, three-level approach to work redesign aimed at optimizing the allocation of registered nursing expertise. These recommendations should be understood as practices to evaluate through local implementation, rather than as effects demonstrated by the present observational study.

### 4.1. Protect Registered Nursing Expertise

Healthcare organizations should first conduct local, task-level workflow audits before changing skill mix or redistributing activities. These audits should identify not only how nurses spend their time, but also the clinical judgment, assessment, accountability, and continuity requirements associated with each activity. Particular attention should be given to protecting registered nurse time for comprehensive assessment, clinical surveillance, recognition of deterioration, complex medication management, care planning and evaluation, therapeutic communication and education, clinical coordination, and supervision of the overall care process. This approach is consistent with the Enacted Scope of Practice model, which distinguishes between the activities nurses are educated and authorized to perform and the activities they are actually able to enact in everyday practice [20,25]. Importantly, the relevant organizational objective is not simply to maximize direct-care minutes. Time spent away from the bedside on complex care coordination, clinical interpretation, discharge planning, or multidisciplinary communication may represent scope-concordant registered nursing practice.

Skill-mix decisions should also be made cautiously because recent evidence does not establish that delegation is universally beneficial. A 2023 scoping review found few studies measuring patient outcomes after registered nurses delegated care to unlicensed workers and identified a substantial lack of baseline and outcome data for determining effective delegation models [58]. In critical care, a 2024 cohort study found that units with a higher proportion of critical-care registered nurses had lower mortality, delirium, pressure injuries, and length of stay than units with lower registered nurse skill mix [59]. These findings do not imply that support workers cannot contribute safely, but they reinforce the need to protect RN-delivered assessment, surveillance, and clinical decision-making.

### 4.2. Redistribute Activities According to Competence

Organizations should assess staff activities according to the competence required, the patient’s clinical condition, the level of accountability involved, and the supervision necessary, not according to professional title alone. Routine and predictable activities may be suitable for appropriately trained support personnel when patient needs, competency requirements, professional regulation, and supervision arrangements permit.

The introduction of the Nursing Assistant role in Italy creates an opportunity to evaluate skill-mix redesign. Comparable roles exist internationally, including Assistants in Nursing (AIN) in Australia [26], Certified Nursing Assistants (CNA) in the United States [60], and Health Care Assistants (HCA) in the United Kingdom [61], all of which have been shown to reduce the burden of indirect tasks on registered nurses and free time for clinically complex care and therapeutic communication. However, delegation should not be treated as a simple transfer of tasks. An integrative review found that effective delegation depends on the registered nurse’s understanding of the assistant’s role and scope of practice, clear job descriptions, communication, and delegation skills [62]. Additionally, evidence also highlights that the effectiveness of skill-mix models depends on clear task boundaries, structured delegation protocols, and shared responsibility frameworks, ensuring that support roles do not merely shift workload but contribute to enhancing nurses enacted scope of practice and the overall quality of care [20,26].

Before implementation, organizations should therefore establish: competency profiles for the Nursing Assistant and registered nurse roles; explicit criteria identifying activities that may and may not be delegated; patient acuity and stability thresholds; documentation and escalation requirements; supervision and reassessment responsibilities; communication procedures between registered nurses and support personnel; and mechanisms for monitoring missed care, adverse events, and continuity. The released RN time should then be tracked directly. Organizations should determine whether it is redirected toward assessment, surveillance, patient education, therapeutic communication, clinical coordination, or other activities requiring registered nursing expertise. If released time is absorbed by additional administrative work, interruptions, or higher patient assignments, the redesign may redistribute workload without increasing enacted scope of practice.

### 4.3. Redesign Inefficient Processes

Activities that are inefficient should be redesigned before they are delegated. Repetitive documentation, duplicate data entry, excessive searching or movement, inefficient telephone processes, avoidable interruptions, fragmented supply systems, and poorly integrated digital platforms may represent system-level workflow problems rather than appropriate work for another occupational group. Delegating an inefficient process may simply transfer inefficiency from registered nurses to support workers.

Evidence in the literature shows that implementing LEAN within healthcare settings can help reorganize nursing activities, enhance efficiency and care quality [63], staff satisfaction, and probably reduce operational costs [64,65,66]. The evidence should be interpreted cautiously since confirmation on improvements concerning patient health, staff well-being, and cost savings remained limited [65,66]. Nursing-specific examples suggest potential benefits. Quality-improvement projects using Lean methods reported reductions in unfinished nursing care, overtime, and improved patient satisfaction [67] and reduction by 55% in nursing time spent collecting and preparing surgical materials [68]. These studies support evaluating process redesign, but they do not demonstrate that all recovered time will automatically become direct or scope-concordant nursing care.

Digital technologies may also help recover nursing time, but implementation should be evaluated for both benefits and unintended workload. For example, barcode medication administration may improve safety when well implemented [69], but it can also disrupt workflow and increase workload when systems are poorly integrated [70,71]. Similarly, informatics systems, such as electronic health records, can enhance information accessibility and care coordination [72], improve documentation time, workflow efficiency, staff satisfaction, and patient interaction; however, objective evidence remains limited [73,74]. Technology is not universally beneficial: its impact depends on usability, system integration, training, and organizational support, and some technologies may initially increase workload or generate alert fatigue if poorly implemented [75].

Importantly, while technology can optimize processes and reduce time spent on administrative and technical activities, it cannot replace the relational, interpretive, and empathic dimensions of nursing, such as clinical judgment, emotional support, and therapeutic communication, areas in which human presence remains essential. The time dedicated to direct patient care and building relationships with the patient, as well as the time spent with the team, remains irreplaceable and fundamental to ensuring quality care and addressing the need for humanization in healthcare [76].

### 4.4. Distinguish Rest from Workflow Loss

Time-motion findings should also distinguish legitimate rest and recovery from avoidable workflow loss. Breaks, brief pauses, and informal recovery periods should not automatically be classified as wasted nursing time, because sustained clinical performance depends on nurses having opportunities to recover during demanding work. By contrast, time lost through unnecessary searching, repeated interruptions, missing supplies, duplicated communication, or poorly designed information systems represents a potential target for redesign. Local workflow audits should therefore classify observed time into at least three categories: clinically necessary nursing work; necessary recovery and legitimate rest; and potentially avoidable workflow loss. This distinction is important because an intervention that eliminates all non-patient care time may increase fatigue and workload rather than improve care. The purpose of redesign should be to remove avoidable inefficiency while preserving the conditions required for safe and sustainable practice.

### 4.5. Evaluation of Redesign Pilots

Before organizations conclude that redistribution or redesign has been beneficial, pilot interventions should include baseline measurement, a defined implementation period, and post-intervention evaluation. Pilot evaluations should include measurements on patient safety, missed care, workload, nurse well-being, patient experience, and continuity of care outcomes. This outcome set is necessary because improvements in efficiency or direct-care time alone may conceal deterioration in continuity, safety, or nurse well-being [60,61]. Likewise, evidence on delegation remains limited, particularly regarding whether delegated care improves patient outcomes [58]. Healthcare organizations should therefore conduct local observational workflow audits before changing skill mix, assess candidate activities according to competence and accountability, protect RN time for complex assessment and surveillance, and redesign inefficient administrative and logistical processes before transferring them to another occupational group. Any redesign should distinguish legitimate rest from avoidable workflow loss and should measure how released RN time is actually used.

From an Enacted Scope of Practice perspective, therefore, the desired organisational outcome is not simply more bedside minutes. A nurse spending more time at the bedside performing tasks that do not require registered nursing competence does not necessarily represent fuller scope enactment. Conversely, time spent away from the bedside coordinating complex care, interpreting clinical information, planning discharge, or communicating with the multidisciplinary team may represent highly scope-concordant nursing practice. The organisational challenge is to ensure that registered nursing expertise is deployed where clinical judgement, professional accountability, and relational competence provide the greatest value.

These considerations may be particularly relevant during the transition into professional practice, when newly qualified nurses move from the student role to the registered nurse role and must reconcile formal scope of practice, professional role expectations, and organisational demands. Among Generation Z nursing students, perceiving the nurse as responsible for patient care was associated with a lower likelihood of choosing nursing as a first-choice career, while some students expressed concerns about assuming such responsibility [77]. From a role-theoretical perspective, these findings may reflect the importance of clear role expectations and supported role transition in the progressive enactment of professional responsibilities. Organisational environments that provide role clarity, appropriate supervision, and opportunities to develop autonomy may therefore facilitate the progressive enactment of nurses’ scope of practice as professional competence and accountability develop.

**Strengths and limitations**

This study has several strengths. Its multicentre observational design provides direct evidence of how registered nurses allocate their working time in routine clinical practice. The inclusion of medical and surgical units from hospitals located in Northern, Central, and Southern Italy increased the heterogeneity of participating contexts and provided insights across different healthcare organizations. Observing complete morning and afternoon shifts over three weeks also allowed nursing activities to be captured across different phases of the working day and reduced the influence of isolated events. Furthermore, the study provides a detailed characterization of nursing work by examining activity categories, frequency, and duration, offering a more comprehensive description of nursing workflow than broad workload indicators alone.

Some limitations should be acknowledged when interpreting the findings. First, the sampling strategy may limit generalizability. Hospital and unit participation was based on voluntary participation, and the included hospitals were teaching institutions; therefore, findings may not fully represent other healthcare settings in Italy. Units were purposely selected within participating hospitals, and the number of units contributed by each hospital was not equal. In addition, only medical–surgical units were included, while specialized settings and other clinical environments may present different patterns of nursing work. Nurse participation was also voluntary, and only nurses who agreed to be observed were included. Although the observation strategy aimed to maximize variability across units and shifts, nurse identifiers were not collected; therefore, the exact number of unique nurses observed and the frequency of repeated observations could not be fully determined. Repeated observations occurred only when alternative participants were unavailable and were minimized to preserve variability in the observation sample. Nevertheless, repeated observations of the same nurse may have resulted in greater representation of individual work patterns and reduced the independence of observations. This may represent a potential source of sampling bias and should be considered when interpreting the findings. Future studies should consider collecting coded identifiers to allow assessment of repeated observations and potential clustering effects.

Second, direct observation introduces potential measurement-related limitations. Although standardized observation procedures, training, pilot testing, and supervision were implemented, a formal inter-observer reliability assessment was not performed. Therefore, differences in observer interpretation, classification, and recording of activities cannot be completely excluded. As data collection relied on direct observation and real-time recording, measurements were partly dependent on the observer’s attention, interpretation, and accuracy in identifying and timing activities. Brief, overlapping, or rapidly changing activities may therefore have been missed or differently classified, introducing potential observer-related measurement bias. Observer-related bias may also have occurred because observers were generally assigned to specific institutions, potentially introducing some overlap between observer characteristics and organizational context. Because observer classification and timing constituted a central component of the measurement process, the absence of formal inter-observer reliability testing limits our ability to quantify the consistency of activity classification and timing across observers. Consequently, some of the observed variability in activity patterns, particularly across institutions where different observers were assigned, may partly reflect observer-related measurement variability rather than differences in nursing work. Future studies should formally assess inter-observer reliability using standardized observation scenarios or independent parallel observations.

Furthermore, the Hawthorne effect cannot be excluded, as nurses may have modified their behaviour because they were aware of being observed. Other factors influencing nursing workflow, including staffing levels, nurse-to-patient ratios, patient acuity, and case mix, were not collected; therefore, differences between hospitals or units cannot be attributed solely to organizational characteristics. Only one nurse was observed per shift, which may not fully represent team-level activity patterns. Other professionals involved in care were not observed, limiting interpretation of task distribution across the wider healthcare team. The reported data-cleaning information does not separately quantify missing or incomplete records, implausible durations, or extreme values, limiting assessment of their potential influence on the results.

Third, the extended data collection period (February 2021–June 2024) represents a further limitation. During this period, healthcare organizations may have experienced changes in staffing availability, care processes, documentation systems, and organizational models. Differences observed between hospitals or units should therefore be interpreted cautiously, as they may reflect both organizational characteristics and temporal heterogeneity across the study period. Similarly, observation periods were selected in collaboration with nurse managers to identify routine clinical activity. Although predefined criteria were used to exclude periods characterized by reduced activity, holidays, increased absenteeism, or unusual organizational conditions, this approach does not represent random temporal sampling and may have underrepresented periods of particularly high workload or workforce strain. In addition, weekends and night shifts were not included; therefore, findings should not be generalized to these periods, during which nursing workflows may differ. Future studies should consider longitudinal sampling across different workload conditions to better capture variability in nursing time allocation.

Finally, the interpretation of findings requires caution. The Enacted Scope of Practice framework was used as an interpretive lens rather than as a formal activity-classification system. Therefore, the study identifies patterns of nursing time allocation and potential areas warranting organizational evaluation but does not establish whether individual activities are unnecessary, delegable, inappropriate, or outside registered nursing scope. Similarly, the organizational and non-patient care categories include heterogeneous activities with different levels of professional responsibility; therefore, broad categories should not be interpreted as inherently suitable for activity redistribution. Although the activity taxonomy was developed through literature-informed procedures, expert review, and pilot testing in clinical settings, formal content validity assessment and inter-rater reliability testing were not performed.

**Future research**

Future studies should extend these findings by examining nursing time allocation across larger and more diverse samples, including non-teaching hospitals, different healthcare settings, and a wider range of clinical contexts. Research should include additional shifts, particularly night and weekends, consider multiple nurses within the same unit, and other members of the healthcare team to better capture variability in nursing work patterns. Incorporating contextual variables such as staffing levels, nurse-to-patient ratios, patient acuity, case mix, documentation systems, technological infrastructure and organizational characteristics would help clarify the organisational factors influencing nursing time allocation.

Methodological development is also needed. Future studies should further validate observational nursing activity taxonomies, establish formal inter-observer reliability, and examine temporal variation in nursing work across different periods and organizational contexts. In addition, research should move beyond broad activity categories and incorporate task-level classification approaches to assess the competencies, clinical judgement, and accountability required for individual activities. This would allow a more precise evaluation of how registered nursing expertise is used and where potential opportunities for workforce redesign may exist.

Further research should also investigate whether different patterns of nursing time allocation are associated with patient outcomes, missed nursing care, patient experience, continuity of care, nurse workload, well-being, and retention. Longitudinal and multicentre studies could help determine whether changes in organizational conditions or workforce models influence the allocation of nursing time over time.

Future intervention studies should evaluate competence-based models of work redesign rather than assuming that redistribution of activities will automatically generate benefits. In particular, studies should assess whether structured redistribution of selected activities to Nursing Assistants, healthcare assistants, or administrative staff increases registered nurses’ opportunities to perform high-value professional activities while maintaining safety, accountability, and continuity of care. Such evaluations should include monitoring of how released registered nursing time is redirected and should examine outcomes such as missed care, patient safety indicators, patient experience, workload, nurse well-being, and retention.

Finally, future research should evaluate complementary organizational strategies, including workflow redesign, reduction in avoidable interruptions, optimization of documentation processes, and digital technologies, to determine whether these approaches effectively support registered nurses in practicing to their full scope of competence and have impact in clinical practice.

## 5. Conclusions

This multicentre observational study provides contemporary evidence on how registered nurses allocate their working time across medical and surgical units in Italy. Direct patient care accounted for 45% of recorded activity time and indirect care for a further 24%, while the remaining time included organizational activities and non-patient care time. These findings demonstrate the complexity of contemporary nursing work, which extends beyond bedside interactions and includes multiple activities required to coordinate, document, and sustain care delivery.

Interpreted through the Enacted Scope of Practice framework, the findings suggest that the professional value of registered nursing cannot be understood solely through the amount of time spent at the bedside. Nursing contribution also derives from activities requiring clinical judgement, professional accountability, care coordination, communication, surveillance, and relational competence. The study therefore contributes to a broader understanding of nursing time allocation by shifting attention from workload quantity alone toward how nursing expertise is utilized within clinical workflows. The Enacted Scope of Practice model was used as an interpretive lens rather than as a formal activity-classification tool; therefore, the findings identify potential opportunities for work redesign but do not establish whether individual activities are unnecessary, delegable, or inappropriate for registered nurses.

The findings identify areas that warrant further competency-based assessment and prospective evaluation of workforce organization strategies. Future research should investigate whether specific approaches to task allocation, delegation, workflow redesign, and skill-mix optimization enable registered nurses to devote more time to activities requiring nursing-specific expertise while maintaining safety, continuity of care, and quality outcomes. Such strategies should be evaluated through structured implementation studies rather than assumed to produce beneficial effects based solely on time redistribution.

## Figures and Tables

**Table 1:** Operational definitions of nursing activity categories.

CLASSIFICATION	DESCRIPTION
DIRECT CARE	Nursing activities performed in the presence of the patient and involving direct interaction between the nurse and the patient; encompassing all tasks involving direct patient interaction and hands-on care delivery.
INDIRECT CARE	Patient-oriented nursing activities performed in the patient’s absence and not requiring direct nurse-patient contact; consisting of patient-related tasks performed away from the bedside such as documentation, care planning, coordination, medication preparation.
ORGANISATIONAL ACTIVITIES	Nursing activities aimed at ensuring the appropriate delivery of direct and indirect care interventions. Although essential to the organisation and effectiveness of care, they do not involve direct nurse-patient contact; including unit management tasks, meetings, supply management, and administrative duties, telephone calls.
NON-PATIENT CARE TIME	A heterogeneous category comprising personal time, waiting, interruptions, movement within the ward, and time outside the ward. Inclusion in this category does not establish that an activity is non-productive, avoidable, or suitable for redistribution.

**Table 2:** **Time Fraction Distribution of Nursing Activities.**

**A. Overall**	**Direct Care (%)**	**Indirect Care (%)**	**Organizational Activities (%)**	**Non-Patient Care (%)**
**Overall**	45%	24%	10%	21%
**B. Hospitals**	**Direct Care (%)**	**Indirect Care (%)**	**Organizational Activities (%)**	**Non-Patient Care (%)**
Hospital 1	45%	20%	10%	25%
Hospital 2	42%	19%	18%	21%
Hospital 3	48%	27%	10%	15%
Hospital 4	45%	40%	5%	10%
Hospital 5	43%	23%	6%	28%
**C. Departments and Hospital–Department Groups**	**Direct Care (%)**	**Indirect Care (%)**	**Organizational Activities (%)**	**Non-Patient Care (%)**
**Surgery, all hospitals**	41%	28%	10%	21%
Surgery, Hospital 1	44%	20%	11%	25%
Surgery, Hospital 3	35%	41%	9%	15%
Surgery, Hospital 4	45%	40%	5%	10%
Surgery, Hospital 5	37%	23%	7%	33%
**Medicine, all hospitals**	51%	17%	11%	21%
Medicine, Hospital 1	47%	21%	6%	26%
Medicine, Hospital 2	42%	19%	18%	21%
Medicine, Hospital 3	65%	8%	13%	14%
Medicine, Hospital 5	46%	24%	5%	25%

Notes: Percentage of cumulative recorded activity duration allocated to each category. (**A**) Overall distribution across all observation periods. (**B**) Distribution by hospital. (**C**) Distributions by department type (surgery or medicine), overall and within hospitals. Each percentage uses the cumulative duration of all activities included in the corresponding overall sample or stratum as its denominator; overlapping activity durations are included. Labels show whole-number percentages. The aggregation procedure is described in Section 2.5.

**Table 3:** **Activity Frequency Distribution in Nursing Workflow.**

**A. Overall**	**Direct Care (%)**	**Indirect Care (%)**	**Organizational Activities (%)**	**Non-Patient Care (%)**
**Overall**	49%	25%	7%	20%
**B. Departments**	**Direct Care (%)**	**Indirect Care (%)**	**Organizational Activities (%)**	**Non-Patient Care (%)**
Surgery, all hospitals	46%	26%	7%	21%
Medicine, all hospitals	53%	22%	6%	19%
**C. Hospital–Department Groups**	**Direct Care (%)**	**Indirect Care (%)**	**Organizational Activities (%)**	**Non-Patient Care (%)**
Surgery, Hospital 1	46%	24%	7%	24%
Surgery, Hospital 3	45%	32%	8%	15%
Surgery, Hospital 4	49%	25%	14%	12%
Surgery, Hospital 5	45%	21%	5%	30%
Medicine, Hospital 1	46%	27%	5%	23%
Medicine, Hospital 2	42%	33%	12%	14%
Medicine, Hospital 3	73%	8%	5%	14%
Medicine, Hospital 5	47%	24%	3%	26%
**D. Hospitals**	**Direct Care (%)**	**Indirect Care (%)**	**Organizational Activities (%)**	**Non-Patient Care (%)**
Hospital 1	46%	25%	6%	23%
Hospital 2	42%	33%	11%	14%
Hospital 3	56%	22%	7%	14%
Hospital 4	49%	25%	14%	12%
Hospital 5	46%	23%	4%	27%

**Notes: Percentage of recorded activity events in each category, independent of duration.** (**A**) Overall distribution. (**B**) Distributions by department type (surgery or medicine). (**C**) Hospital-specific distributions within each department type. (**D**) Overall distributions by hospital. Each percentage uses the total number of activity records included in the corresponding analysis or stratum as its denominator. HbH denotes hospital-by-hospital comparisons. The panels provide descriptive comparisons; no uncertainty intervals are shown.

**Table 4:** **Mean Activity Duration (Unit Time) by category.**

**A. Overall**	**Direct Care (s)**	**Indirect Care (s)**	**Organizational Activities (s)**	**Non-Patient Care (s)**
**Overall**	130 s	130 s	215 s	145 s
**B. Departments**	**Direct Care (s)**	**Indirect Care (s)**	**Organizational Activities (s)**	**Non-Patient Care (s)**
Surgery, all hospitals	125 s	145 s	190 s	140 s
Medicine, all hospitals	135 s	110 s	255 s	150 s
**C. Hospital–Department Groups**	**Direct Care (s)**	**Indirect Care (s)**	**Organizational Activities (s)**	**Non-Patient Care (s)**
Surgery, Hospital 1	120 s	100 s	215 s	125 s
Surgery, Hospital 3	115 s	185 s	160 s	145 s
Surgery, Hospital 4	230 s	390 s	95 s	220 s
Surgery, Hospital 5	150 s	205 s	300 s	200 s
Medicine, Hospital 1	120 s	90 s	135 s	130 s
Medicine, Hospital 2	180 s	105 s	295 s	260 s
Medicine, Hospital 3	135 s	150 s	355 s	150 s
Medicine, Hospital 5	140 s	140 s	260 s	135 s
**D. Hospitals**	**Direct Care (s)**	**Indirect Care (s)**	**Organizational Activities (s)**	**Non-Patient Care (s)**
Hospital 1	115 s	95 s	195 s	125 s
Hospital 2	180 s	100 s	295 s	260 s
Hospital 3	120 s	180 s	220 s	145 s
Hospital 4	225 s	390 s	95 s	220 s
Hospital 5	145 s	160 s	280 s	160 s

Notes: Mean recorded duration per activity event, expressed in seconds, within each activity category. (**A**) Overall comparison. (**B**) Comparison by department type (surgery or medicine). (**C**) Hospital-specific comparisons within each department type. (**D**) Overall comparison by hospital. Each mean is calculated as cumulative recorded duration divided by the number of contributing events in the corresponding category and stratum, as defined in Section 2.5. HbH denotes hospital-by-hospital comparisons. The panels provide descriptive comparisons; no uncertainty intervals are shown.

**Table 5:** **Internal composition of the direct patient care activity category.**

Subcategory	Activity Records (% of Category)	Cumulative Duration (% of Category)
Circulatory system care	6.96%	8.99%
Gastrointestinal system care	2.14%	3.03%
Respiratory system care	1.61%	1.95%
Urinary system care	3.10%	3.11%
Hygiene care	4.35%	9.44%
Wound care/Dressing changes	3.49%	4.69%
Patient mobilization	3.59%	5.61%
Vital signs monitoring	28.55%	18.69%
Invasive procedures care	0.61%	1.48%
Admission/Discharge/Patient communication	8.79%	14.01%
Bed making	2.12%	2.81%
Medication administration	34.37%	25.65%
Swab collection	0.31%	0.47%
Emergency care	0.01%	0.06%
**Total**	**100.00%**	**100.00%**

Notes: The proportion of activity records for each subcategory was calculated by dividing the number of records in that subcategory by the total number of records within the overarching category. Similarly, the proportion of recorded time was calculated by dividing the cumulative duration of each subcategory by the total recorded duration of its overarching category. Percentages were calculated using the direct patient care category as the denominator and do not represent proportions of total observed nursing time.

**Table 6:** **Internal composition of the indirect patient care activity category.**

Subcategory	Activity Records (% of Category)	Cumulative Duration (% of Category)
Nursing documentation/review	18.47%	18.69%
Verbal patient handover	5.02%	15.91%
Dietary care planning	1.29%	2.30%
Medical record review/data entry	27.59%	18.18%
Communication	17.72%	23.21%
Multidisciplinary ward round	1.41%	4.71%
Medication preparation	28.51%	17.00%
**Total**	**100.00%**	**100.00%**

Notes: The proportion of activity records for each subcategory was calculated by dividing the number of records in that subcategory by the total number of records within the overarching category. Similarly, the proportion of recorded time was calculated by dividing the cumulative duration of each subcategory by the total recorded duration of its overarching category. Percentages were calculated using the indirect patient care category as the denominator and do not represent proportions of total observed nursing time.

**Table 7:** **Internal composition of the organizational activity category.**

Subcategory	Activity Records (% of Category)	Cumulative Duration (% of Category)
Delegation	32.00%	26.55%
Managerial/organizational tasks	21.26%	37.65%
Incoming telephone calls	13.27%	7.33%
Isolation-related activities	12.00%	4.72%
Outgoing telephone calls	11.71%	5.68%
Training	9.69%	17.48%
Computer work	0.07%	0.59%
**Total**	**100.00%**	**100.00%**

Notes: The proportion of activity records for each subcategory was calculated by dividing the number of records in that subcategory by the total number of records within the overarching category. Similarly, the proportion of recorded time was calculated by dividing the cumulative duration of each subcategory by the total recorded duration of its overarching category. Percentages were calculated using the organisational care category as the denominator and do not represent proportions of total observed nursing time.

**Table 8:** **Internal composition of the non-patient care category.**

Subcategory	Activity Records (% of Category)	Cumulative Duration (% of Category)
Interruptions	45.24%	35.39%
Time outside the ward	0.85%	3.98%
Personal time	5.14%	15.38%
Waiting	1.05%	2.47%
Ward movement	47.72%	42.78%
**Total**	**100.00%**	**100.00%**

Notes: The proportion of activity records for each subcategory was calculated by dividing the number of records in that subcategory by the total number of records within the overarching category. Similarly, the proportion of recorded time was calculated by dividing the cumulative duration of each subcategory by the total recorded duration of its overarching category. Percentages were calculated using the non-patient care category as the denominator and do not represent proportions of total observed nursing time.

## Data Availability

Data are available from the corresponding author upon reasonable request.

## Supplementary Materials

The following supporting information can be downloaded at: https://www.mdpi.com/article/10.3390/nursrep16100372/s1, Supplementary File SA: Classification of Nursing Activities; Supplementary File SB: Distribution of Nursing Activities Within the Four Activity Classifications Across Units and Hospitals; Supplementary File SC: Strobe checklist.

## Public Involvement Statement

No public involvement in any aspect of this research.

## Guidelines and Standards Statement

This manuscript was drafted against the STROBE (Strengthening the Reporting of Observational Studies in Epidemiology) for observational research.

## Use of Artificial Intelligence

ChatGPT GPT 6 Luna (OpenAI) was used during manuscript preparation to support English-language translation and editing, improve grammar, clarity, conciseness, and assist with the rephrasing of selected sections of the manuscript. The tool was also used to support the refinement of textual descriptions of the manuscript based exclusively on theoretical frameworks and scientific literature provided by the authors. Artificial intelligence was not used for data collection, data generation, statistical analysis, or manipulation of study results. All AI-assisted content was critically reviewed, verified, and revised by the authors, who take full responsibility for the accuracy, interpretation, originality, and final content of the manuscript.

## Abbreviations

The following abbreviations are used in this manuscript:

ASCOP	Actual Scope of Nursing Practice
STROBE	Strengthening the Reporting of Observational Studies in Epidemiology
AIN	Assistants in Nursing
CNA	Certified Nursing Assistants
HCA	Health Care Assistants
JD-R	Job Demands–Resources
