# Supplementary material for: How Is Registered Nursing Time Used? A Multicentre Observational Study of Nursing Activities, Scope of Practice, and Opportunities for Workforce Redesign in Medical–Surgical Wards in Italy
Source: Nurs Rep. 2026 Oct 20;16(10):372. doi: 10.3390/nursrep16100372 (PMC13708415; doi:10.3390/nursrep16100372)
Supplement: Supplementary file 1 [file nursrep-16-00372-s001.zip › Supplementary File SA.pdf]

Supplementary File SA. Classification of Nursing Activities

CLASSIFICATION	DESCRIPTION
**DIRECT CARE**	Nursing activities performed in the presence of the patient and involving direct interaction between the nurse and the patient.
**INDIRECT CARE**	Patient-oriented nursing activities performed in the patient's absence and not requiring direct nurse-patient contact.
**ORGANISATIONAL ACTIVITIES**	Nursing activities aimed at ensuring the appropriate delivery of direct and indirect care interventions. Although essential to the organisation and effectiveness of care, they do not involve direct nurse-patient contact.
**NON-PATIENT CARE TIME**	Activities not strictly related to nursing care, intended for the physical and mental well-being of the healthcare worker, and non-productive periods that do not provide a direct benefit to either the nurse or the patient.

**QUESTIONNAIRE**<br>**Grouping nursing activities**	**ITEM**<br>**Nursing activities**	**CLASSIFICATION**
**VITAL SIGNS**	Blood pressure (BP)<br>Heart rate (HR)<br>Capillary blood glucose (Dtx/HGB)<br>Body temperature<br>SpO2<br>Respiratory rate (RR)<br>Pain<br>Central venous pressure (CVP)<br>Monitor: A - placement/removal/battery replacement; B - reading/assessment of parameters	**DIRECT CARE**
**SWAB COLLECTION**	Swab collection (oral, nasal, rectal, vaginal, wound)	**DIRECT CARE**
**MEDICATION ADMINISTRATION**	Oral<br>Intravenous: A - intermittent/short infusion; B - continuous infusion; C - monitoring; D - removal/discontinuation<br>Enteral: A - placement/initiation; B - replenishment; C - monitoring; D - removal/discontinuation<br>Subcutaneous<br>Inhaled/O2 therapy<br>Intramuscular<br>A - ocular; B - transdermal; C - other<br>A - assessment; B - communication	**DIRECT CARE**
**CIRCULATORY SYSTEM**	Electrocardiogram<br>Venous blood sampling (preparation + performance)<br>Blood sampling from a central line/arterial line<br>Peripheral intravenous catheter (PIVC) insertion<br>PIVC: A - removal; B - dressing; C - flushing; D - assessment<br>A - PICC dressing; B - CVC dressing<br>A - PICC flushing; B - CVC flushing<br>A - PICC removal; B - CVC removal<br>A - PICC assessment; B - CVC assessment<br>Blood transfusion (entire process, excluding blood sampling)<br>Communication<br>Assessment	**DIRECT CARE**
**RESPIRATORY SYSTEM**	Simple airway suctioning<br>Tracheostomy suctioning<br>Tracheostomy dressing<br>Sputum collection<br>A - initiation/placement of O2 therapy; B - O2 therapy monitoring<br>NIV/CPAP management and monitoring<br>Education on respiratory exercises	**DIRECT CARE**

	A - physical examination; B - assessment; C - communication	
**URINARY SYSTEM**	Placement of: A - urinary catheter; B - bedpan; C - urinal<br>Removal of: A - urinary catheter; B - bedpan; C - urinal<br>Urinary catheter irrigation<br>BAG REPLACEMENT<br>Emptying: A - urinary catheter bag; B - urinal; C - jug<br>Urine testing: A - sample from urinary catheter; B - routine urine sample; C - urine culture<br>Urostomy management<br>Initiation of bladder irrigation<br>Emptying bladder irrigation drainage<br>Intermittent urinary catheterisation<br>A - physical examination; B - assessment; C - communication	**DIRECT CARE**
**DIGESTIVE SYSTEM**	Assistance with feeding<br>Enteral feeding<br>Evacuative enema (preparation + administration)<br>Sorbiclis<br>A - rectal examination; B - manual evacuation<br>A - tube insertion; B - tube check<br>A - stool sample collection; B - stool assessment<br>A - nasogastric tube (NGT) insertion; B - NGT management<br>A - nausea management; B - vomiting management<br>Stoma management<br>Food diary<br>Physical examination<br>A - assessment; B - communication	**DIRECT CARE**
**HYGIENE**	Partial hygiene care<br>Full hygiene care<br>A - oral care<br>B - dressing<br>C - hair removal (trichotomy)<br>A - assessment; B - communication	**DIRECT CARE**
**MOBILISATION**	A - bed-to-chair transfer; B - chair-to-bed transfer<br>A - repositioning; B - mobilisation in bed<br>A - assistance to the bathroom<br>B - restraint<br>C - bandaging<br>D - anti-embolism stockings<br>E - ice application<br>A - assessment; B - communication	**DIRECT CARE**
**BED MAKING**	Partial bed making<br>Simple bed making<br>Occupied bed making<br>Bed preparation (splint, bed cradle, pressure-relieving device)<br>Stripping the bed<br>A - assessment; B - communication	**DIRECT CARE**
**WOUND CARE/DRESSINGS**	Materials preparation<br>Surgical wound dressing<br>Dressing check<br>Pleurevac check<br>A - Pleurevac dressing; B - Pleurevac removal<br>Drain check<br>A - drain dressing; B - drain removal<br>Complex wound dressing<br>VAC therapy: A - management; B - dressing; C - check<br>Pressure injury dressing<br>A - assessment; B - communication	**DIRECT CARE**
**INVASIVE PROCEDURES**	A - materials preparation; B - assistance during the procedure<br>Patient preparation<br>Care of an unstable patient<br>Emergency care<br>A - assessment; B - communication	**DIRECT CARE**

**ADMISSION / COMMUNICATION**	Patient admission/welcome<br>PATIENT DISCHARGE<br>PATIENT TRANSFER<br>Nursing documentation/form<br>Conversation with the patient<br>Patient/family education<br>Ensuring patient comfort	**DIRECT CARE**
**MEDICATION PREPARATION**	Intermittent/short intravenous therapy<br>Continuous intravenous therapy<br>Enteral therapy<br>Oral therapy<br>Total medication preparation time	**INDIRECT CARE**
**Other INDIRECT ACTIVITIES**	Writing nursing handover notes for each patient<br>Review of the nursing record<br>Review of the medical/clinical record<br>Recording data in the clinical record<br>Calculation: A - fluid balance; B - drain output<br>Verbal handover for each patient<br>Completion of risk-prevention forms (falls, pressure injuries, malnutrition)<br>Dietary planning	**INDIRECT CARE**
**COMMUNICATIONS**	Ward round<br>Consultations: A - nursing colleagues; B - nurse manager<br>Consultations with physicians<br>Consultations with other professionals	**INDIRECT CARE**
**TELEPHONE / INCOMING CALLS**	Call from physician<br>Call from service/department<br>Call from admissions office<br>External call<br>Call from pharmacy	**ORGANISATIONAL ACTIVITY**
**TELEPHONE / OUTGOING CALLS**	Call to specimen transport service<br>Call to patient transport service<br>Call to pharmacy (medications/supplies/requests)<br>Call to internal services (cleaning, maintenance, linen service, engineering, wards, other services)<br>Call to external services (ambulance)<br>Call to physician<br>Call to admissions office	**ORGANISATIONAL ACTIVITY**
**COMPUTER WORK**	Management of own time/attendance record<br>Email	**ORGANISATIONAL ACTIVITY**
**TRAINING**	Students<br>Newly hired staff<br>Nursing colleagues<br>Healthcare assistants (OSS)<br>Physicians	**ORGANISATIONAL ACTIVITY**
**ISOLATION**	A - initiation of isolation precautions; B - discontinuation of isolation precautions<br>Donning personal protective equipment (PPE)<br>Equipment disinfection<br>Communication	**ORGANISATIONAL ACTIVITY**
**DELEGATION**	Student<br>Healthcare assistant (OSS)<br>Nurse<br>Nurse manager<br>Physician	**ORGANISATIONAL ACTIVITY**
**MANAGERIAL / ORGANISATIONAL TASKS**	Ordering medications/supplies<br>Expiry-date checks<br>Restocking ward areas<br>Sorting/distributing supplies<br>Cleaning and tidying ward areas<br>Quality checks (glucometer, emergency trolley, morphine stock)<br>Checking shift schedules and attendance<br>Management of online procedures	**ORGANISATIONAL ACTIVITIES**
**MEDICATION TROLLEY PREPARATION**	A - trolley cleaning<br>B - trolley restocking<br>C - waste disposal	**ORGANISATIONAL ACTIVITY**
**INTERRUPTION**	Telephone<br>Physician	**NON-PATIENT CARE TIME**

	Nurse/healthcare assistant (OSS)<br>Student<br>Family member<br>Patient<br>Call bell<br>Transport/catering/physiotherapy staff<br>Nurse manager<br>Ward intercom<br>Visitor	
**TIME OUTSIDE THE WARD**	Transportation or search for materials:<br>Operating room<br>Intensive care unit<br>Service/department<br>Transfer of the patient to another ward	**NON-PATIENT CARE TIME**
**PERSONAL TIME**	Break/toilet<br>Internet use<br>Personal phone use<br>Conversation with colleagues	**NON-PATIENT CARE TIME**
**WAITING**	Waiting time	**NON-PATIENT CARE TIME**
**WARD MOVEMENT**	Searching for colleagues<br>Searching for supplies/materials<br>Searching for the physician<br>Moving from one ward area to another<br>Moving towards the patient	**NON-PATIENT CARE TIME**
