# Supplementary material for: How Is Registered Nursing Time Used? A Multicentre Observational Study of Nursing Activities, Scope of Practice, and Opportunities for Workforce Redesign in Medical–Surgical Wards in Italy
Source: Nurs Rep. 2026 Oct 20;16(10):372. doi: 10.3390/nursrep16100372 (PMC13708415; doi:10.3390/nursrep16100372)
Supplement: Supplementary file 1 [file nursrep-16-00372-s001.zip › Supplementary File SB.pdf]

Supplementary file SB. Distribution of Nursing Activities Within the Four Activity Classifications Across Units and Hospitals

The tables below provide a detailed breakdown of the nursing activities included within the four predefined activity classifications: direct care nursing activities, indirect care nursing activities, organisational nursing activities, and non-patient care nursing activities.
For each participating medical and surgical unit across the five hospitals, the table reports the mean proportion of observed shift time (%) allocated to each specific activity area within its corresponding classification.

Direct Care Nursing Activities	Circulatory system care	Gastrointestinal system care	Respiratory system care	Urinary system care	Hygiene care	Wound care / Dressing changes	Patient mobilization	Vital signs monitoring	Invasive procedures care	Admission / Discharge/Patient Communication	Bed making	Medication administration	Swab collection	Emergency care
Hospital 1 Surgery Unit 1	4,40%	1,75%	1,38%	3,08%	5,13%	1,03%	4,18%	18,83%	3,11%	19,36%	3,29%	12,87%	0,06%	1%
Hospital 1 Medical Unit 1	4,07%	0,07%	0,00%	0,77%	0,11%	0,80%	0,33%	38,92%	0,05%	4,99%	0,19%	3,15%	0,19%	0%
Hospital 1 Surgery Unit 2	3,57%	1,23%	0,21%	1,06%	1,32%	0,83%	1,00%	18,21%	1,19%	7,34%	1,13%	8,99%	0,27%	0%
Hospital 1 Surgery Unit 3	1,68%	1,31%	0,52%	3,77%	12,07%	9,19%	10,20%	18,36%	0,17%	6,79%	3,75%	9,27%	1,84%	0%
Hospital 1 Medical Unit 2	2,94%	0,79%	0,62%	4,92%	0,64%	0,92%	1,74%	25,52%	0,23%	6,00%	0,21%	13,38%	0,16%	0%
Hospital 2 Medical Unit 3	11,04%	2,74%	2,51%	2,64%	11,10%	4,45%	4,71%	2,88%	2,54%	3,17%	1,67%	13,52%	0,38%	0%
Hospital 3 Medical Unit 4	6,37%	1,03%	3,38%	1,72%	5,60%	3,99%	1,63%	9,10%	1,94%	7,36%	0,26%	26,68%	0,17%	0%
Hospital 3 Surgery Unit 4	15,85%	1,43%	1,81%	3,09%	4,98%	4,12%	7,45%	1,78%	0,08%	2,16%	1,71%	24,49%	0,05%	0%
Hospital 3 Medical	19,35%	0,61%	0,00%	0,45%	11,81%	0,39%	1,36%	0,82%	0,17%	3,15%	2,99%	22,12%	0,35%	0%

Unit 5														
Hospital 3 Surgery Unit 5	0,79%	2,82%	0,75%	1,06%	3,72%	7,65%	5,23%	2,03%	0,00%	1,47%	1,08%	30,39%	0,13%	0%
Hospital 4 Surgery Unit 6	1,94%	2,64%	0,15%	1,78%	10,26%	1,10%	4,64%	4,69%	0,16%	1,50%	2,41%	9,95%	0,03%	0%
Hospital 5 Surgery Unit 7	2,53%	1,72%	0,01%	2,15%	0,76%	2,71%	1,21%	4,10%	0,49%	12,93%	0,93%	9,97%	0,00%	0%
Hospital 5 Medical Unit 6	4,93%	2,29%	3,59%	1,46%	5,09%	1,60%	4,40%	3,32%	1,57%	9,78%	0,74%	19,86%	0,03%	0%

**Indirect Care Nursing Activities**	**Nursing documentation/review**	**Verbal patient handover**	**Dietary care planning**	**Medical record review/data entry**	**Comunication with other healthcare professionals**	**Communication with the nurse manager**	**Communication with nursing colleagues**	**Communication with physicians**	**Multidisciplinary ward round**	**medication preparation: Intermittent intravenous**	**medication preparation: Continuous intravenous**	**medication preparation: Enteral therapy**	**medication preparation: Oral medication**
Hospital 1 Surgery Unit 1	13,28%	7,10%	2,12%	11,38%	1,48%	2,09%	5,16%	5,49%	0,69%	1,12%	2,06%	0,05%	1,85%
Hospital 1 Medical Unit 1	1,55%	3,31%	0,96%	2,19%	0,29%	0,10%	0,58%	0,86%	1,19%	0,27%	0,04%	0,02%	0,57%
Hospital 1 Surgery Unit 2	3,39%	5,13%	1,28%	3,55%	0,62%	0,00%	1,57%	0,21%	0,64%	0,82%	0,49%	0,02%	1,22%
Hospital 1 Surgery Unit 3	7,35%	7,33%	2,26%	8,68%	2,66%	0,54%	2,38%	1,44%	0,30%	1,77%	2,12%	0,26%	1,57%
Hospital 1 Medical Unit 2	4,78%	8,21%	1,33%	8,05%	0,74%	0,20%	1,58%	2,73%	0,00%	8,03%	1,05%	0,09%	1,33%
Hospital 2 Medical	3,07%	3,91%	0,13%	2,30%	0,33%	0,62%	3,19%	2,17%	3,81%	2,10%	2,18%	1,85%	2,28%

Unit 3													
Hospital 3 Medical Unit 4	3,84%	0,64%	0,00%	1,69%	0,30%	1,03%	0,60%	0,96%	2,11%	0,58%	0,51%	0,46%	0,55%
Hospital 3 Surgery Unit 4	20,64%	5,17%	1,11%	11,21%	0,56%	1,77%	4,51%	1,88%	1,27%	2,63%	3,19%	1,78%	1,44%
Hospital 3 Medical Unit 5	1,34%	1,08%	0,00%	3,01%	0,00%	0,00%	1,55%	0,26%	0,43%	0,26%	0,82%	0,24%	0,37%
Hospital 3 Surgery Unit 5	23,42%	9,76%	0,00%	10,54%	2,87%	9,63%	12,48%	4,68%	4,84%	2,26%	0,91%	0,87%	2,02%
Hospital 4 Surgery Unit 6	1,30%	6,04%	0,00%	15,42%	0,54%	0,06%	2,96%	0,57%	0,92%	4,68%	0,64%	0,28%	1,42%
Hospital 5 Surgery Unit 7	5,67%	4,97%	0,27%	4,85%	0,14%	0,06%	2,41%	0,90%	0,00%	2,07%	0,45%	0,13%	2,40%
Hospital 5 Medical Unit 6	7,37%	3,89%	0,19%	3,37%	0,27%	0,76%	1,68%	1,62%	2,42%	3,67%	1,11%	0,37%	3,45%

Organisational Nursing Activities	Quality control checks (glucometer, emergency cart, controlled drugs, expiry dates)	Online administrative procedures	Cleaning, tidying, and restocking of clinical areas and medications	Staff COVID-19 testing	Delegation of activities to:Nurse manager	Delegation of activities to:Registered nurse	Delegation of activities to:Physician	Delegation of activities to:Nurse Assistants	Delegation of activities to:Student	Education and training activities to students, newly hired staff, and healthcare assistants	Isolation organisation: Communication, setup, removal, sanitisation, and donning procedures	Incoming telephone calls:from admissions, pharmacy, physicians, internal services, and external callers	Outgoing telephone calls: to cleaning, maintenance, linen services, engineering, hospital wards, specimen and patient transport, admissions, pharmacy, external services (ambulance), and physicians
Hospital 1 Surgery Unit 1	2,70%	6,42%	13,61%	0,82%	0,36%	1,17%	0,66%	7,03%	0,46%	0,23%	1,70%	2,54%	2,12%
Hospital	0,05%	0,77%	14,43%	0,00%	0,00%	0,02%	0,05%	0,00%	0,00%	0,00%	0,65%	0,92%	2,13%

1 Medical Unit 1													
Hospital 1 Surgery Unit 2	0,96%	3,09%	13,70%	0,14%	0,00%	0,00%	0,10%	0,07%	0,32%	0,00%	0,66%	2,23%	2,10%
Hospital 1 Surgery Unit 3	0,04%	2,69%	13,98%	0,77%	0,00%	0,08%	0,09%	0,27%	0,00%	0,51%	0,92%	4,80%	2,35%
Hospital 1 Medical Unit 2	0,00%	3,14%	13,24%	0,12%	0,00%	0,21%	0,13%	0,22%	0,00%	0,00%	2,12%	1,94%	2,53%
Hospital 2 Medical Unit 3	1,15%	1,34%	13,83%	0	0,06%	0,40%	0,05%	1,10%	2,22%	13,41%	0,70%	4,08%	3,80%
Hospital 3 Medical Unit 4	0,00%	1,79%	13,30%	0	2,05%	0,02%	0,46%	2,53%	0,85%	2,31%	0,57%	0,58%	4,38%
Hospital 3 Surgery Unit 4	0,00%	0,42%	15,42%	0	0,14%	0,87%	0,86%	2,59%	1,42%	1,29%	0,87%	0,95%	2,20%
Hospital 3 Medical Unit 5	0,00%	0,00%	12,88%	0	0,00%	0,00%	0,43%	1,70%	11,21%	0,13%	0,57%	0,61%	1,69%
Hospital 3 Surgery Unit 5	1,59%	1,79%	13,99%	0	0,06%	0,98%	1,80%	0,30%	2,43%	4,88%	1,94%	1,86%	2,38%
Hospital 4 Surgery Unit 6	0,16%	0,73%	12,88%	0	0,00%	0,01%	0,00%	0,84%	0,20%	1,58%	1,30%	0,81%	1,88%
Hospital 5 Surgery Unit 7	1,89%	1,62%	13,09%	0	0,00%	0,00%	0,00%	0,31%	0,00%	1,62%	0,73%	1,82%	2,09%
Hospital 5 Medical Unit 6	0,00%	0,88%	13,34%	0	0,00%	0,11%	0,06%	1,86%	0,97%	0,43%	0,76%	0,92%	1,77%

Non-patient Care Nursing Activities	Waiting time	Interruption	Moving within the ward	Personal time	Time outside the ward
Hospital 1 Surgery Unit 1	1,87%	32,36%	64,21%	2,38%	0,41%
Hospital 1 Medical Unit 1	0,16%	6,52%	8,29%	2,52%	0,73%
Hospital 1 Surgery Unit 2	0,00%	7,06%	13,79%	3,41%	0,92%
Hospital 1 Surgery Unit 3	0,42%	3,61%	8,40%	2,68%	0,98%
Hospital 1 Medical Unit 2	0,60%	4,78%	34,88%	2,61%	0,64%
Hospital 2 Medical Unit 3	4,03%	8,62%	5,75%	8,73%	4,72%
Hospital 3 Medical Unit 4	0,00%	12,29%	12,36%	3,61%	0,30%
Hospital 3 Surgery Unit 4	0,00%	10,33%	15,48%	2,86%	0,00%
Hospital 3 Medical Unit 5	0,00%	0,00%	0,00%	0,00%	0,09%
Hospital 3 Surgery Unit 5	0,00%	5,72%	10,63%	1,94%	0,00%
Hospital 4 Surgery Unit 6	0,79%	2,10%	2,19%	2,38%	1,92%
Hospital 5 Surgery Unit 7	0,35%	7,12%	12,01%	13,26%	2,06%
Hospital 5 Medical Unit 6	0,00%	11,70%	13,99%	4,72%	0,68%
